# Supplementary figures and images for: Prominin 1 and Tweety Homology 1 both induce extracellular vesicle formation
Source: eLife. 2024 Aug 13;13:e100061. doi: 10.7554/eLife.100061 (PMC11405016; doi:10.7554/eLife.100061)

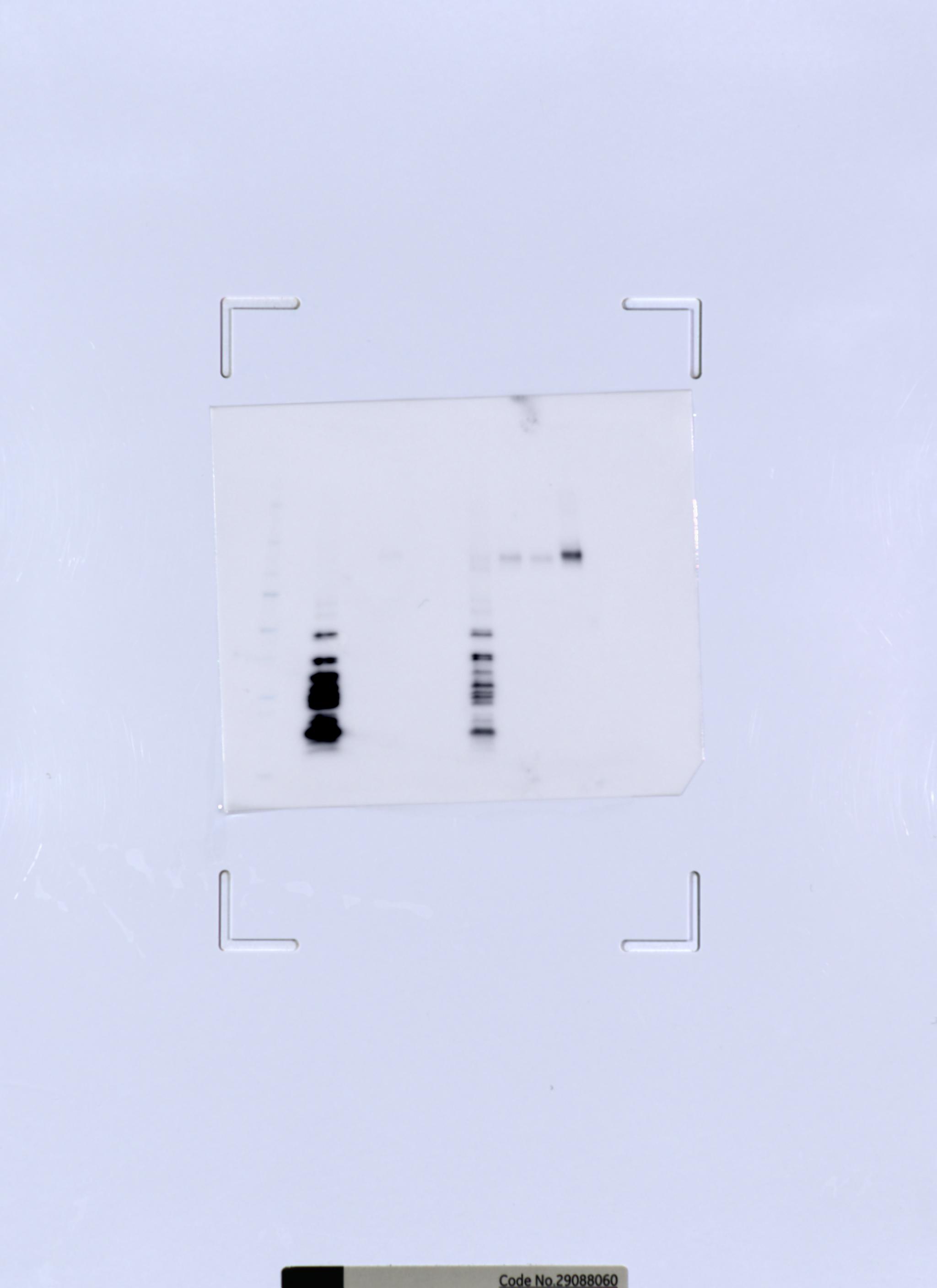

Supplement: Figure 1—source data 1. [file elife-100061-fig1-data1.zip › Figure 1-source data 1/Fig1B_022224-mockvprom 2024.02.22_13.39.42_Ch+Marker.jpg]

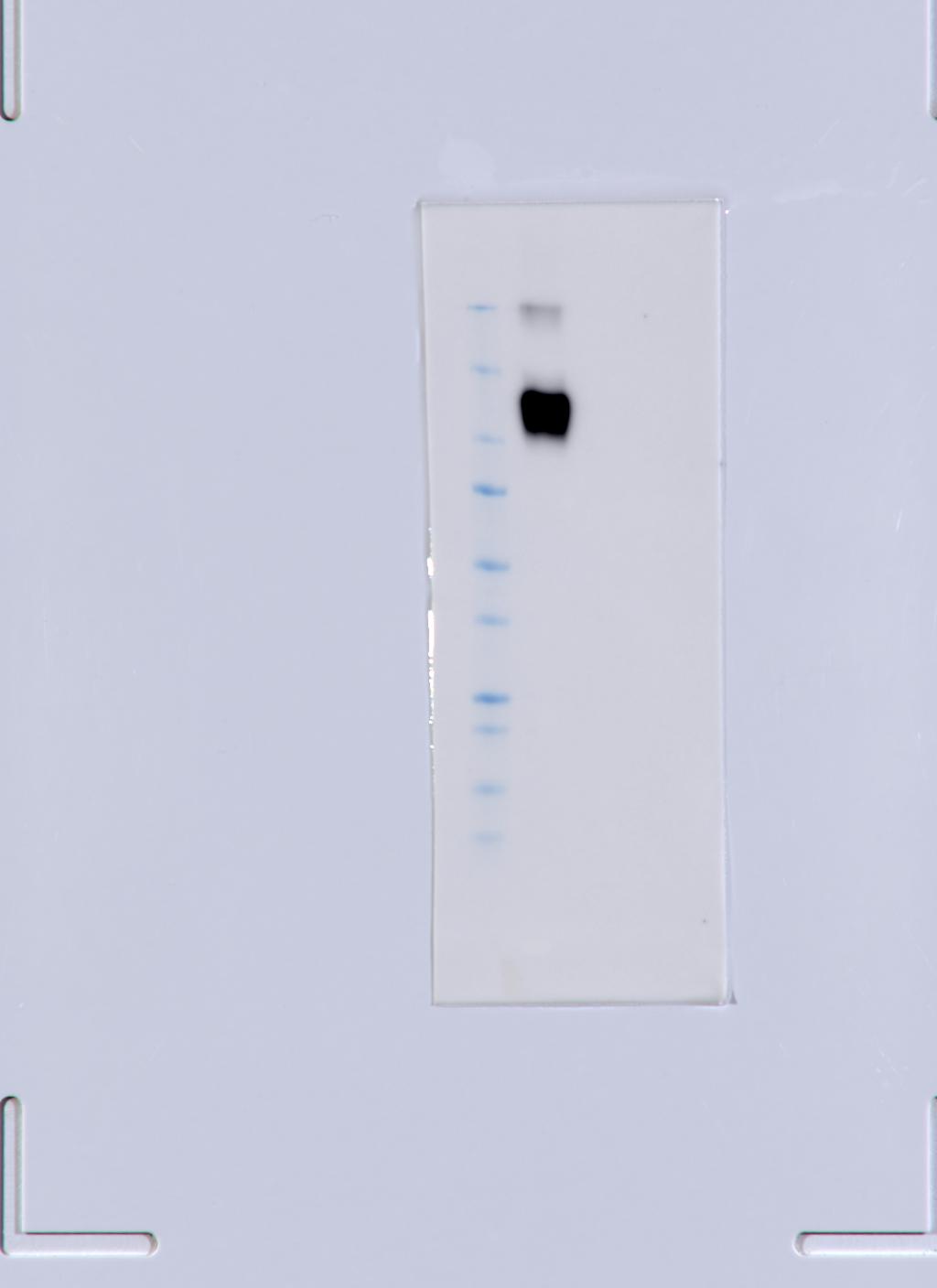

Supplement: Figure 1—source data 1. [file elife-100061-fig1-data1.zip › Figure 1-source data 1/Fig1C_20230904-wt prom1 ac133 2023.09.06_11.25.01_Ch+Marker.jpg]

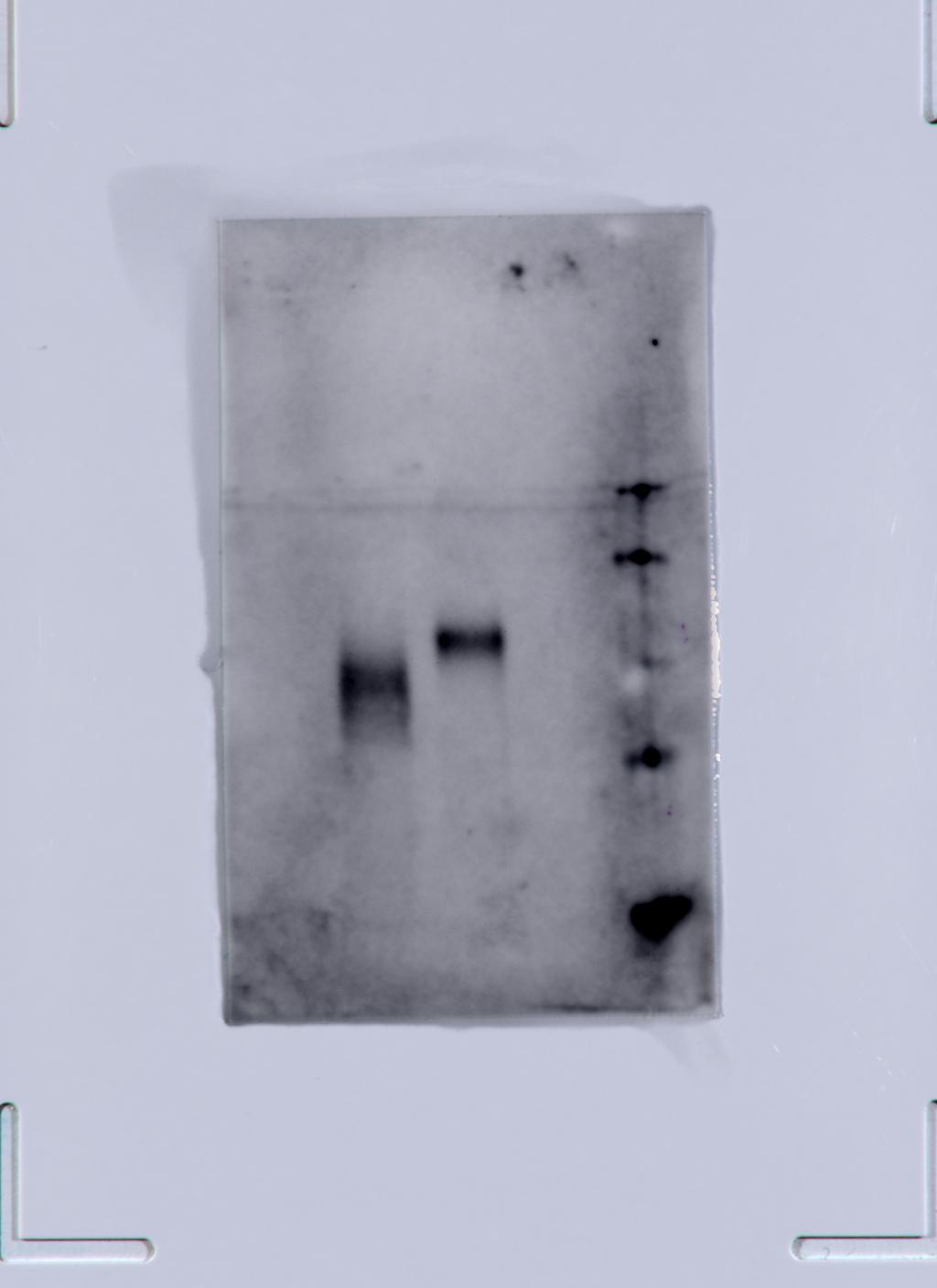

Supplement: Figure 1—source data 1. [file elife-100061-fig1-data1.zip › Figure 1-source data 1/Fig1D_230719-pngaseBcholnorm-aStrep 2023.07.19_13.06.36_Ch+Marker.jpg]

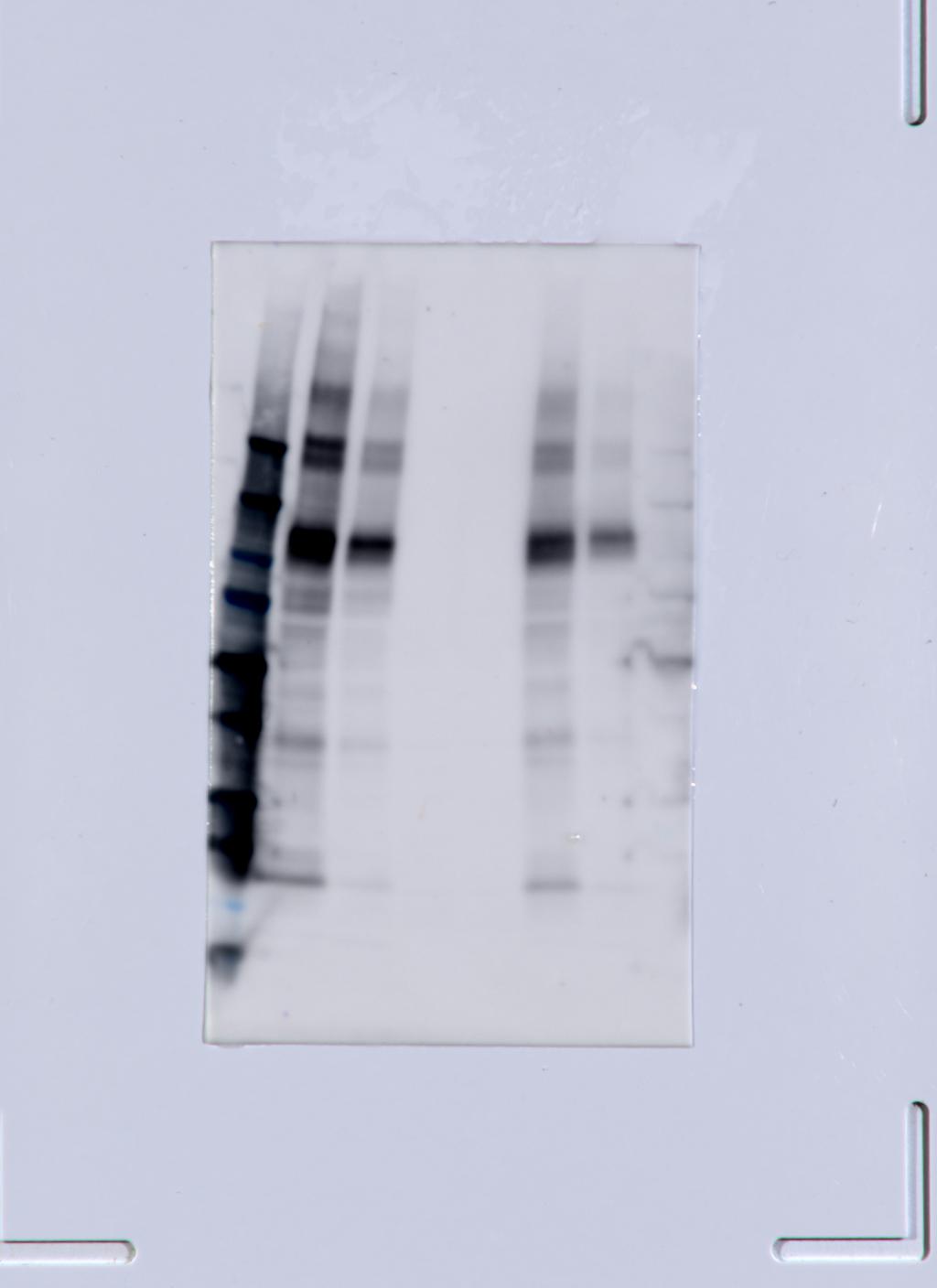

Supplement: Figure 1—source data 1. [file elife-100061-fig1-data1.zip › Figure 1-source data 1/Fig1H_left_220722-prompcdh-antiStrep 2022.07.22_12.06.01_Ch+Marker.jpg]

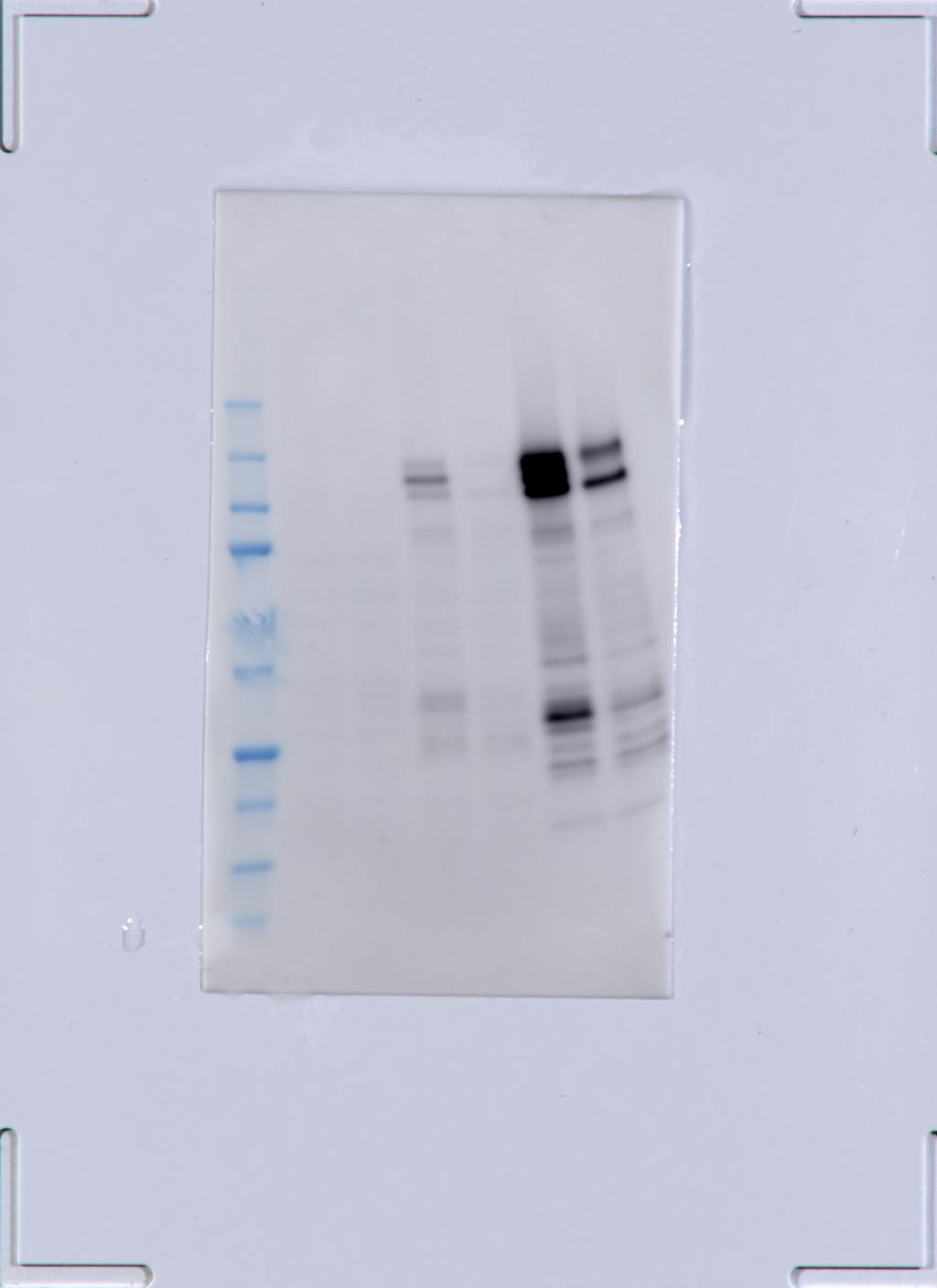

Supplement: Figure 1—source data 1. [file elife-100061-fig1-data1.zip › Figure 1-source data 1/Fig1H_right_220722-prompcdh-antiFlag 2022.07.22_12.08.49_Ch+Marker.jpg]

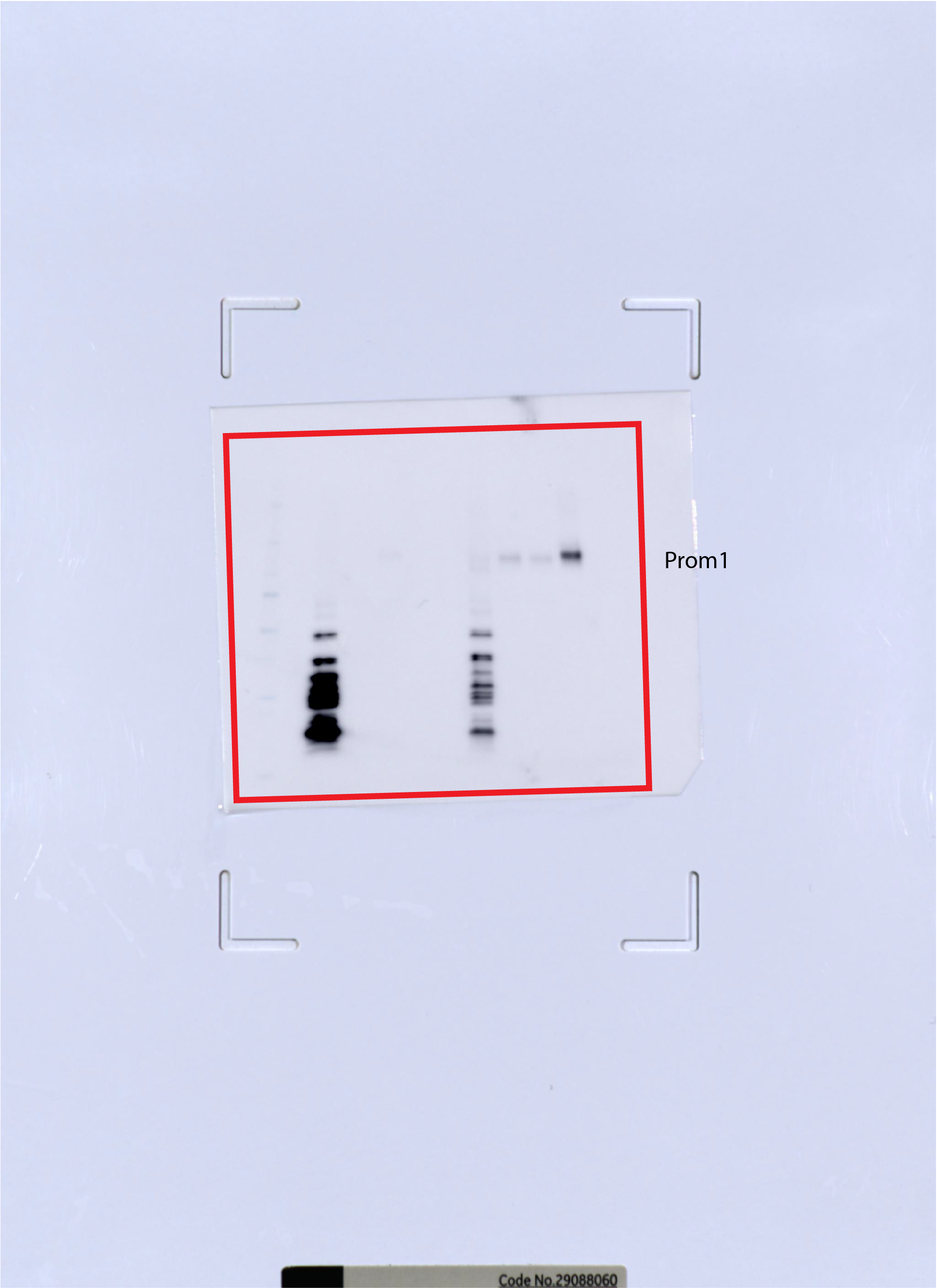

Supplement: Figure 1—source data 2. [file elife-100061-fig1-data2.zip › Figure 1-source data 2/Fig1B_WITH_RED_BOX_labeled.png]

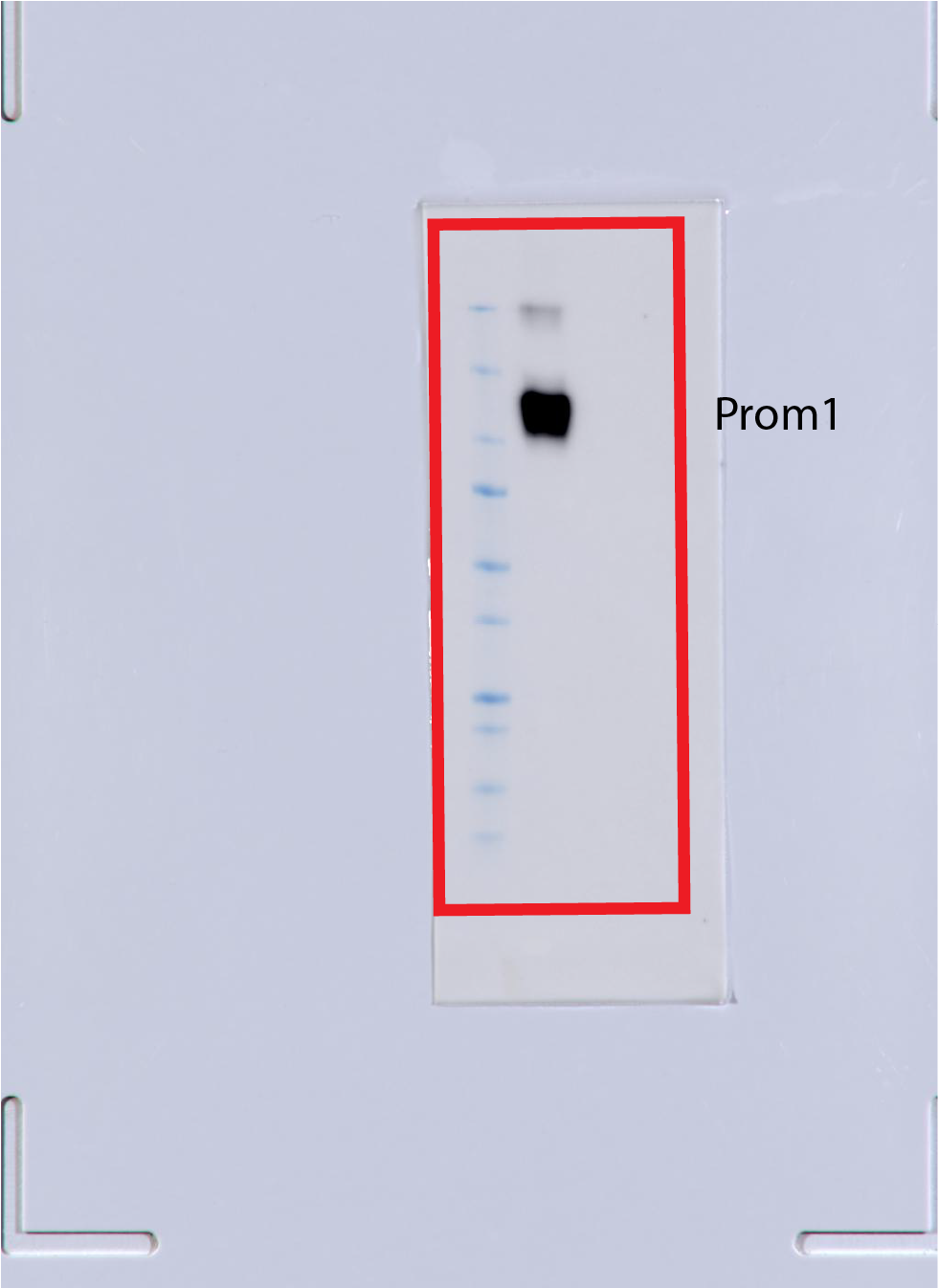

Supplement: Figure 1—source data 2. [file elife-100061-fig1-data2.zip › Figure 1-source data 2/Fig1C_WITH_RED_BOX_labeled.png]

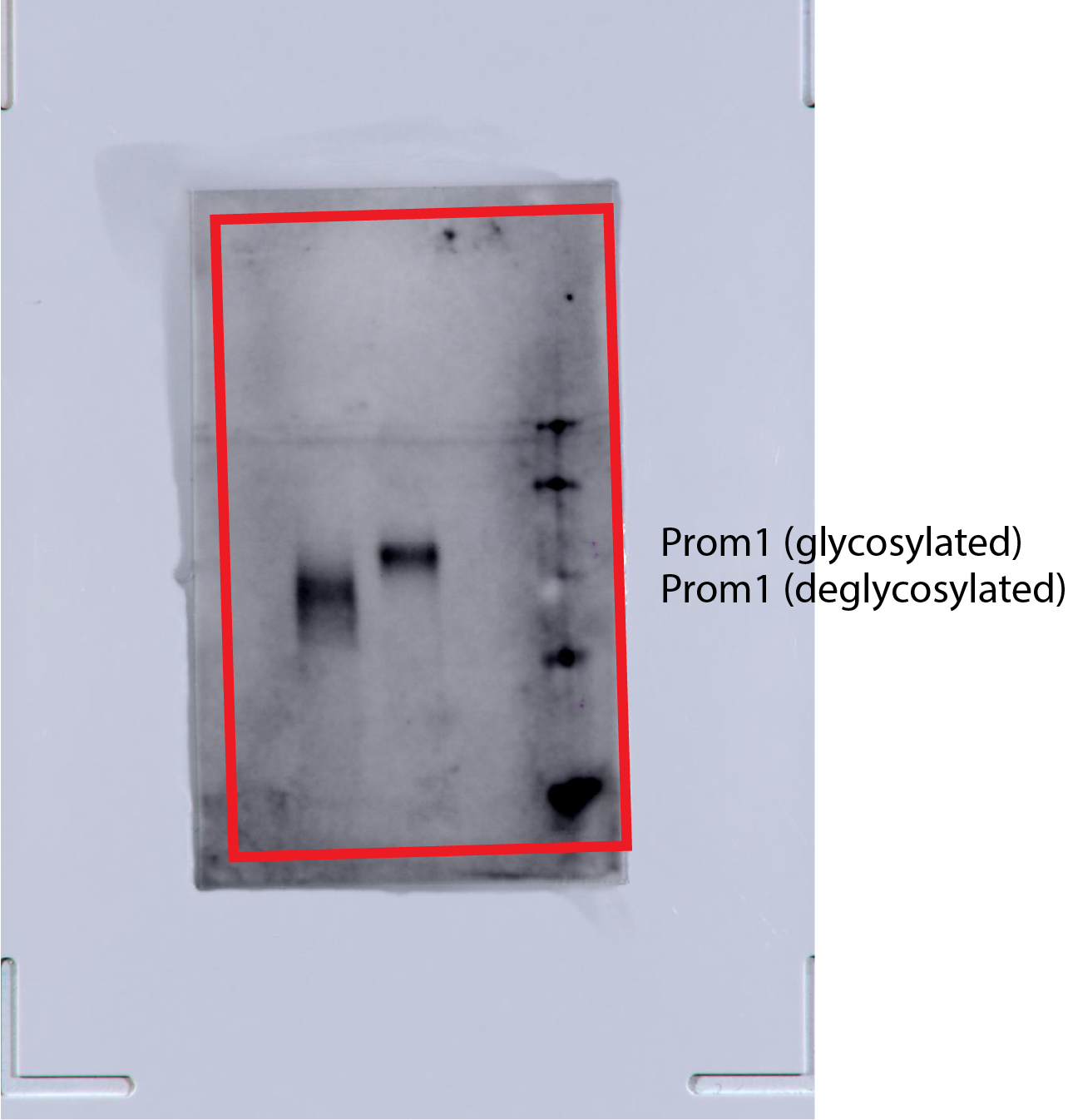

Supplement: Figure 1—source data 2. [file elife-100061-fig1-data2.zip › Figure 1-source data 2/Fig1D_WITH_RED_BOX_labeled.png]

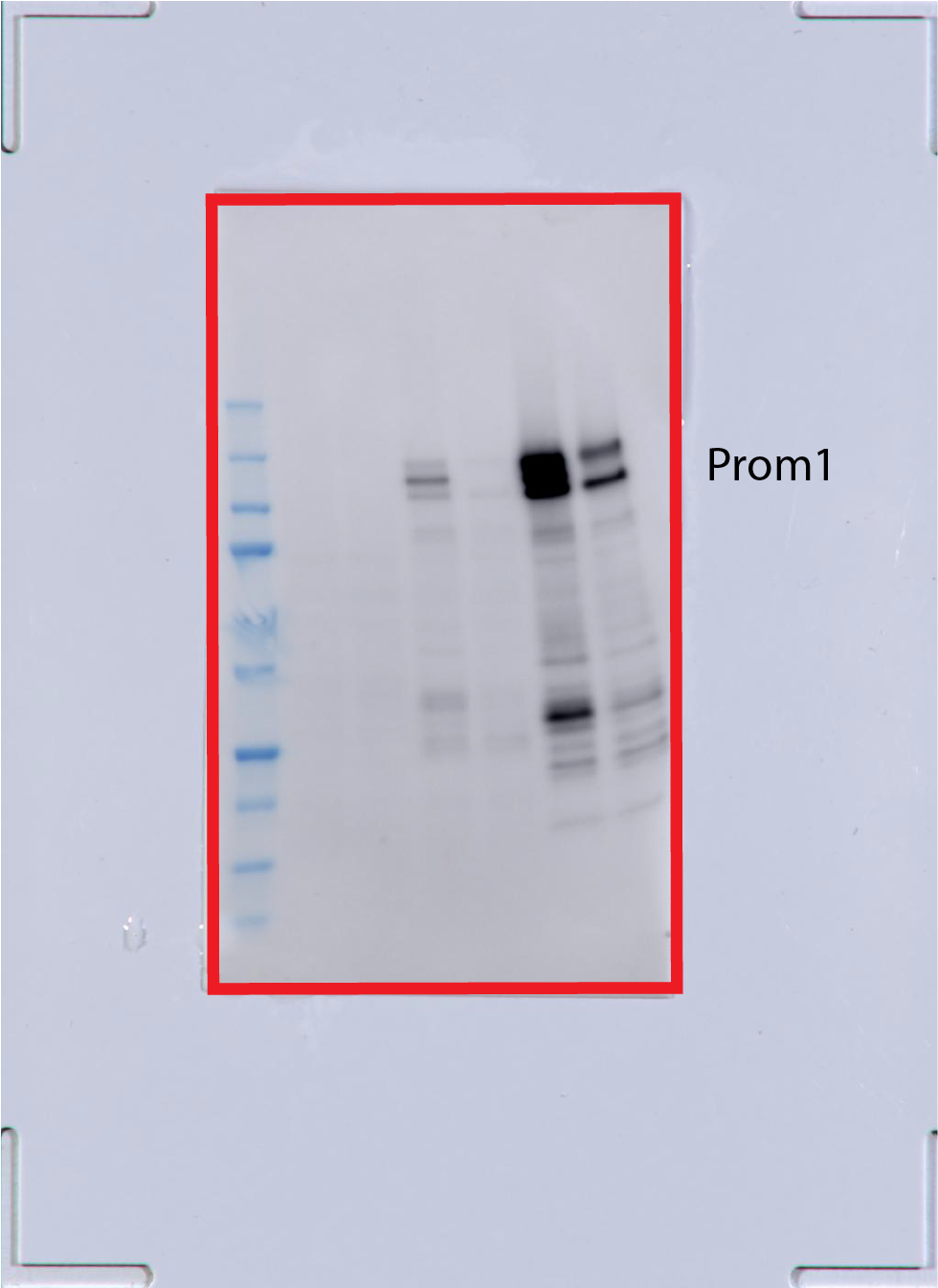

Supplement: Figure 1—source data 2. [file elife-100061-fig1-data2.zip › Figure 1-source data 2/Fig1H_right_WITH_RED_BOX_labeled.png]

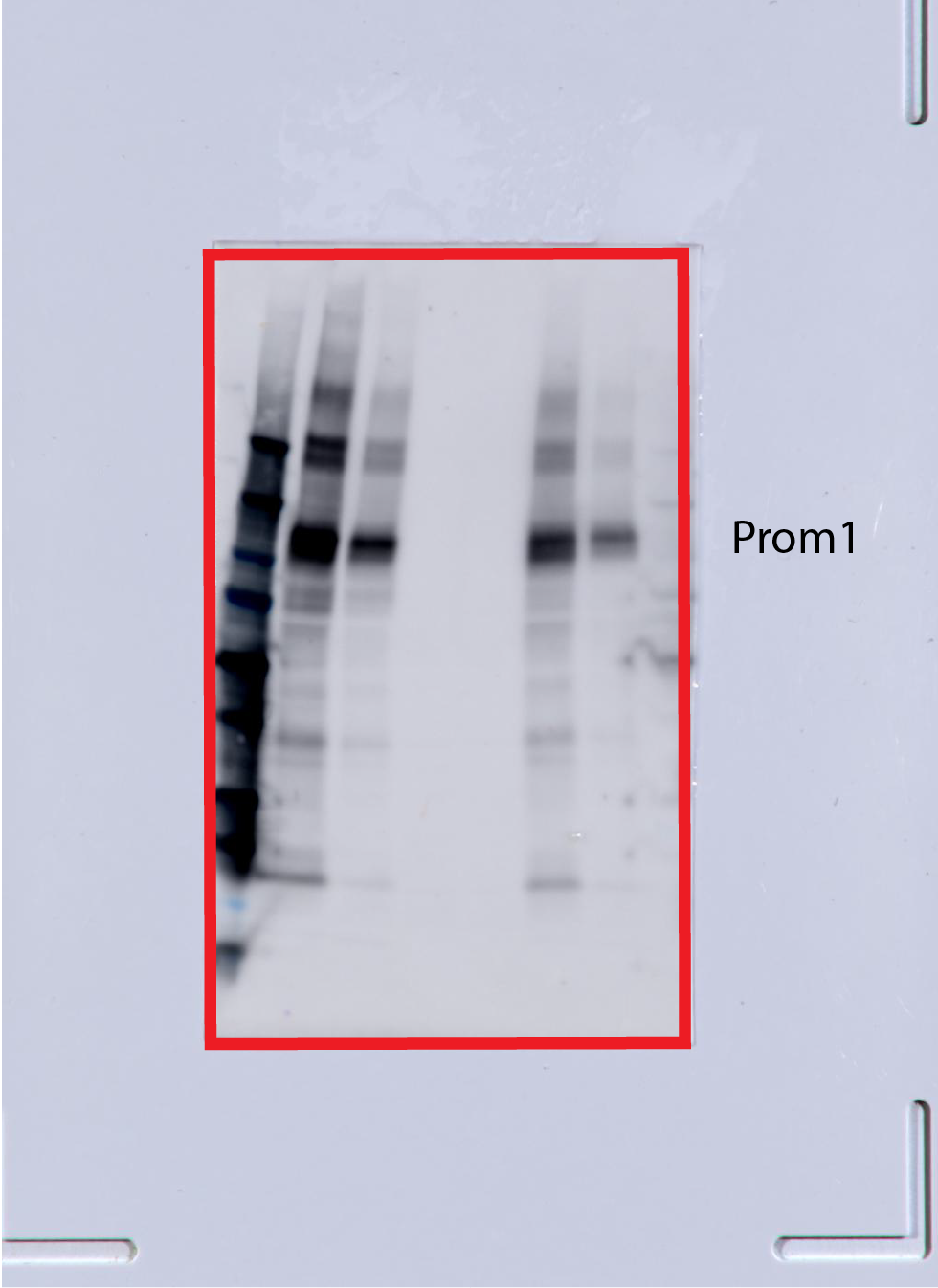

Supplement: Figure 1—source data 2. [file elife-100061-fig1-data2.zip › Figure 1-source data 2/Fig1Hleft_WITH_RED_BOX_labeled.png]

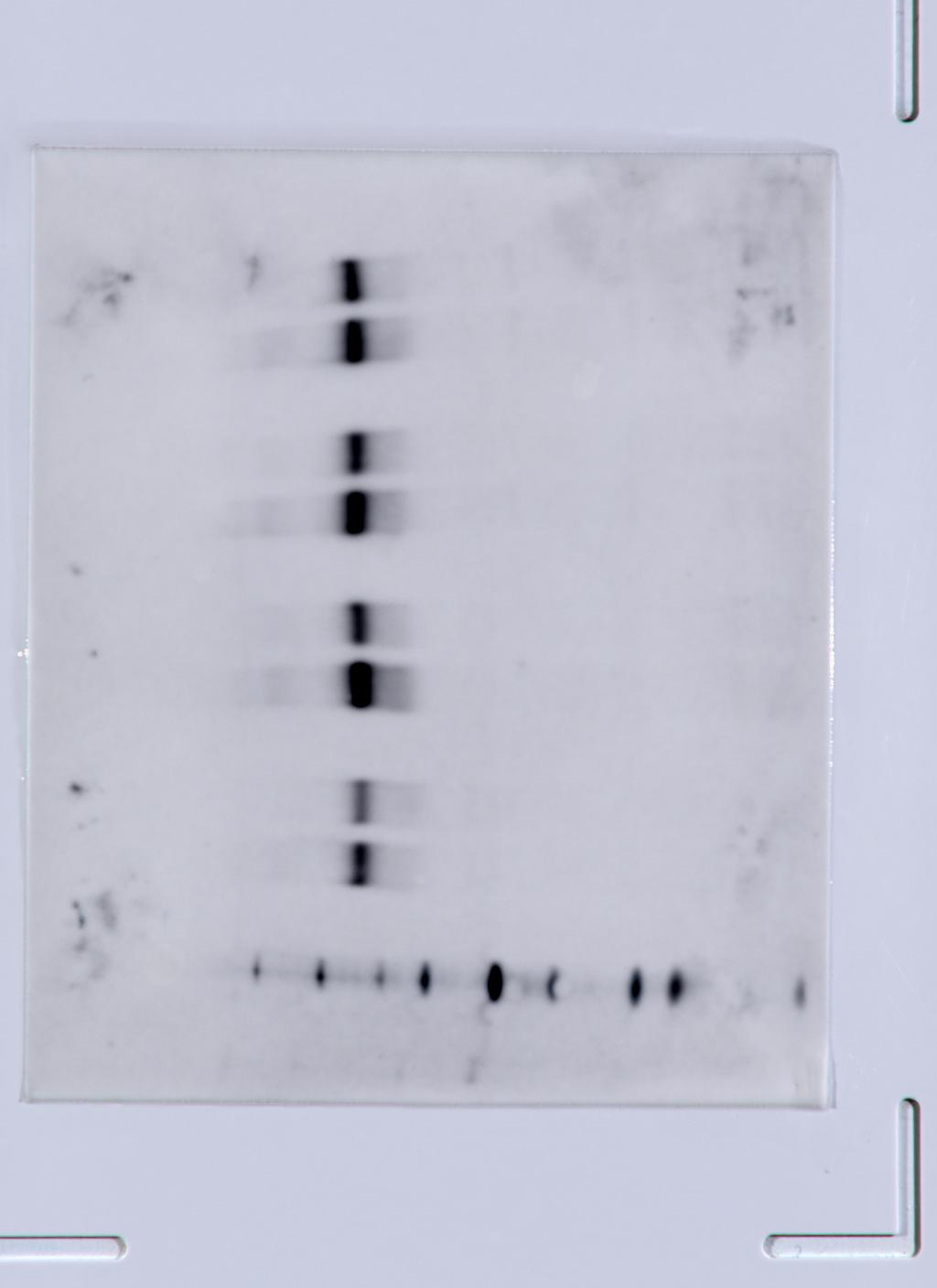

Supplement: Figure 1—figure supplement 1—source data 1. [file elife-100061-fig1-figsupp1-data1.zip › Figure 1-figure supplement 1-source data 1/1A_230719-timaFinal-aStrep 2023.07.19_13.35.20_Ch+Marker.jpg]

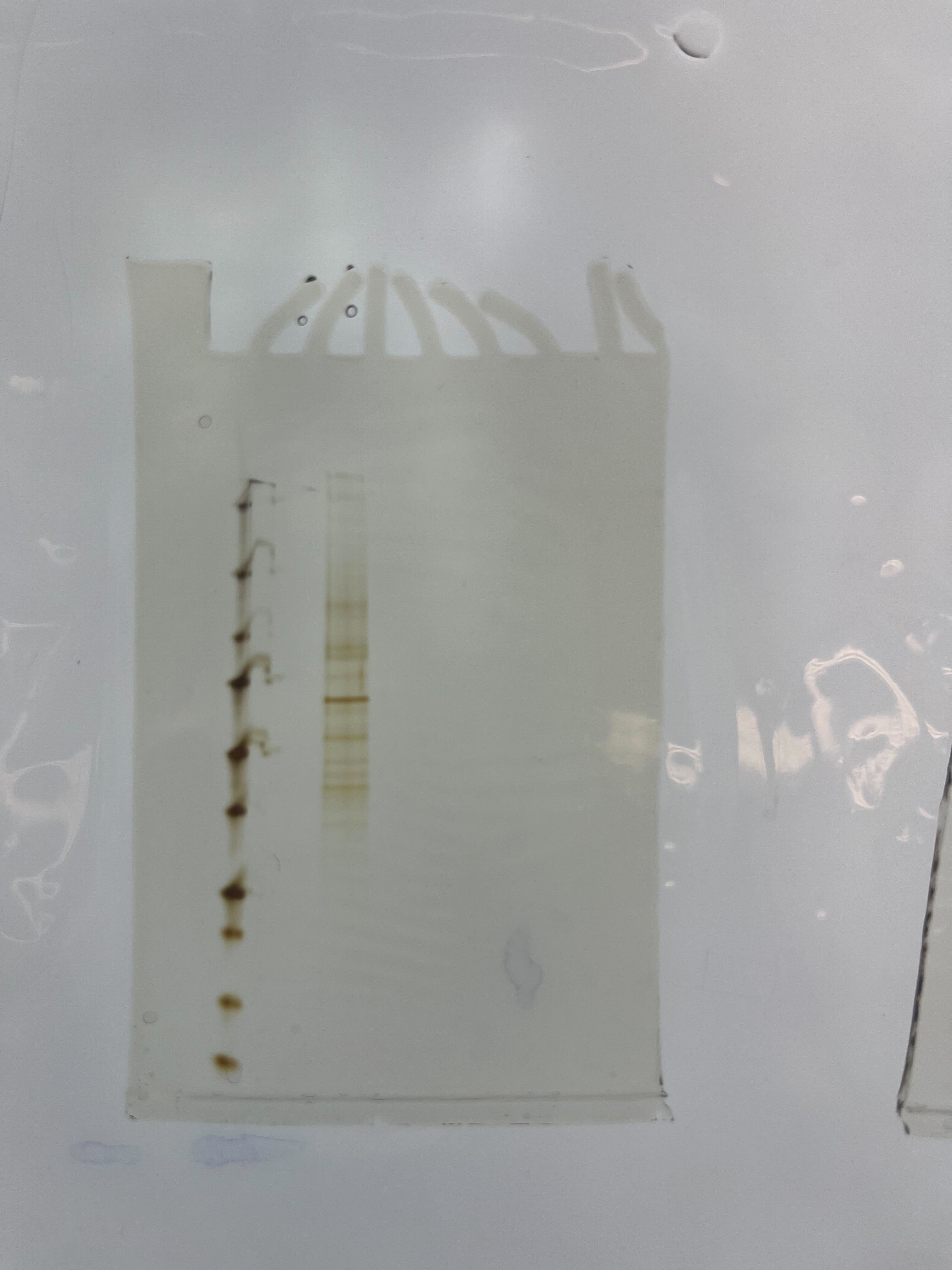

Supplement: Figure 1—figure supplement 1—source data 1. [file elife-100061-fig1-figsupp1-data1.zip › Figure 1-figure supplement 1-source data 1/1B_IMG_4174.jpg]

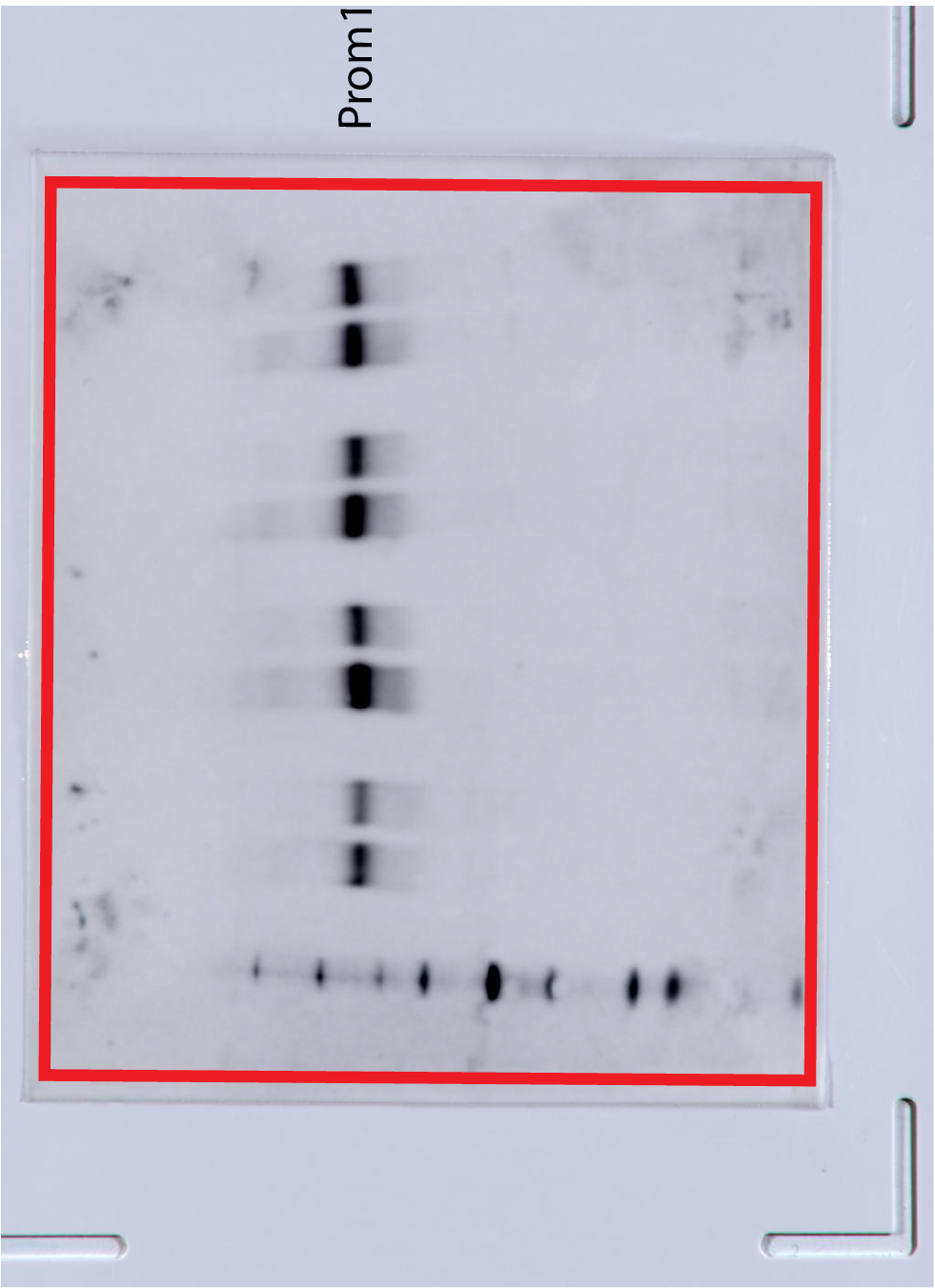

Supplement: Figure 1—figure supplement 1—source data 2. [file elife-100061-fig1-figsupp1-data2.zip › Figure 1-figure supplement 1-source data 2/1A_WITH_RED_BOX_labeled.png]

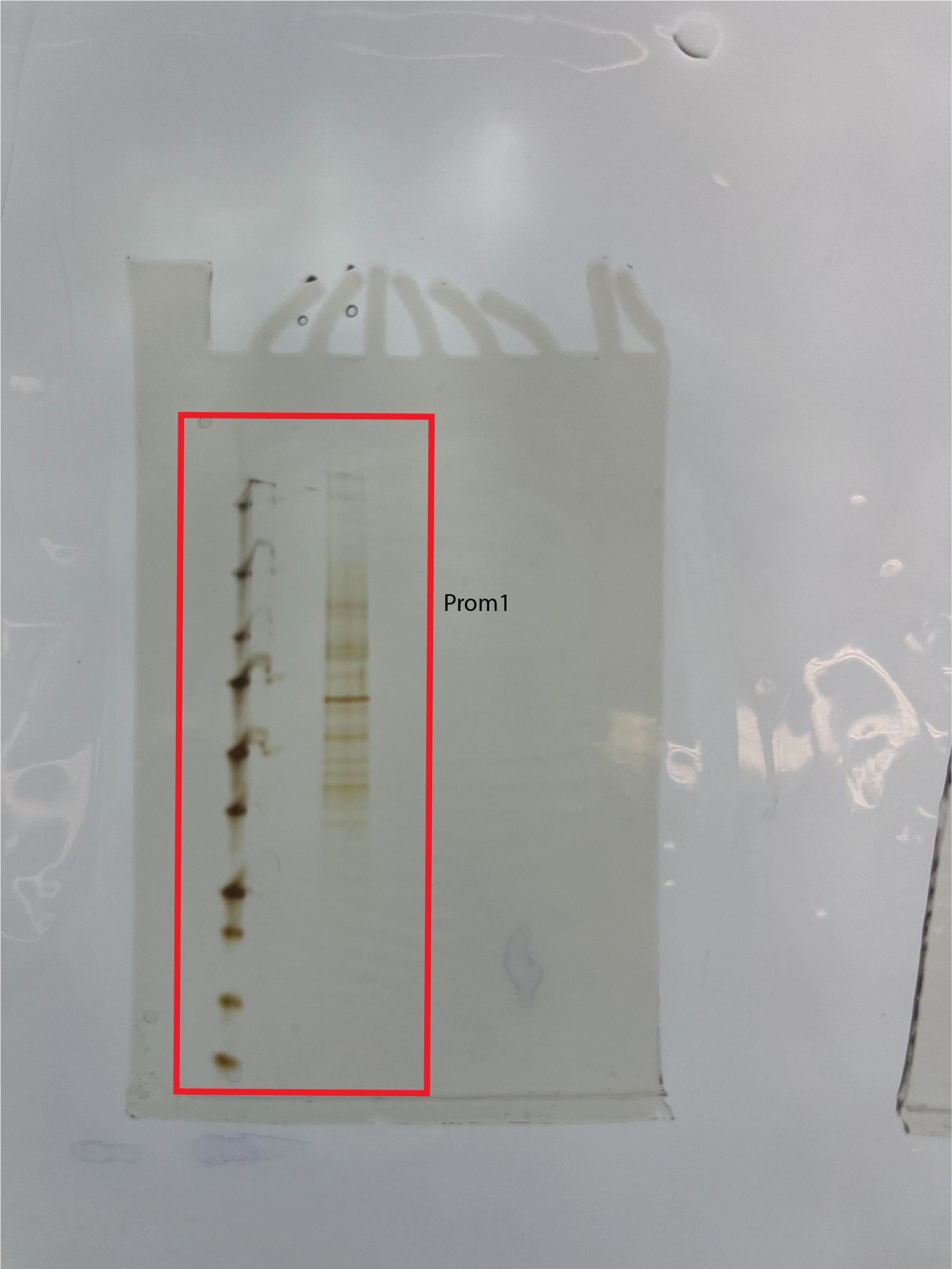

Supplement: Figure 1—figure supplement 1—source data 2. [file elife-100061-fig1-figsupp1-data2.zip › Figure 1-figure supplement 1-source data 2/1B_WITH_RED_BOX_labeled.png]

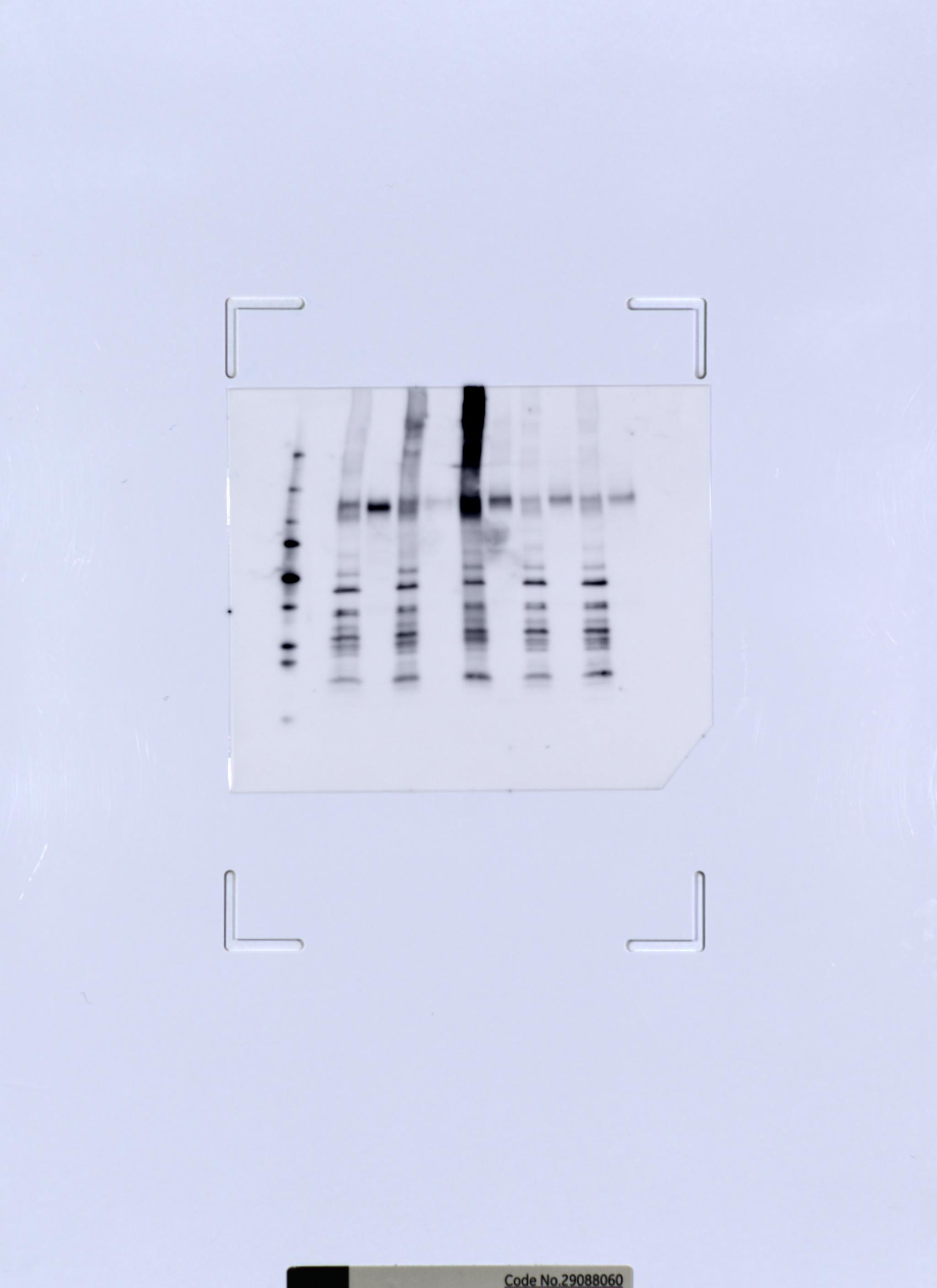

Supplement: Figure 2—figure supplement 4—source data 1. [file elife-100061-fig2-figsupp4-data1.zip › Figure 2ΓÇöfigure supplement 4ΓÇösource data 1/panel1_rep2_021924-bel-mutants-1B 2024.02.19_13.59.01_Ch+Marker.jpg]

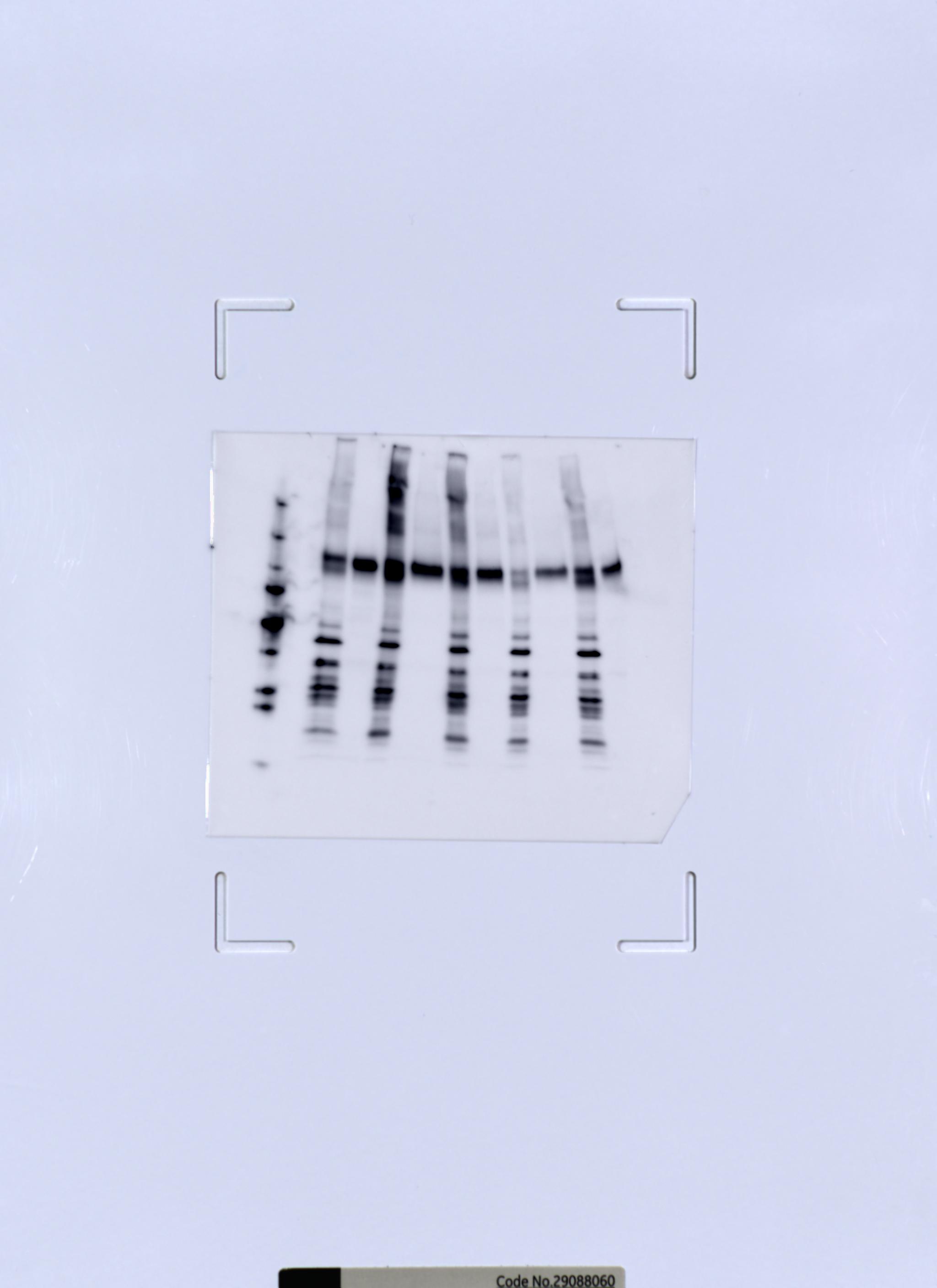

Supplement: Figure 2—figure supplement 4—source data 1. [file elife-100061-fig2-figsupp4-data1.zip › Figure 2ΓÇöfigure supplement 4ΓÇösource data 1/panel1_rep1_021924-bel-mutants-1A 2024.02.19_14.09.59_Ch+Marker.jpg]

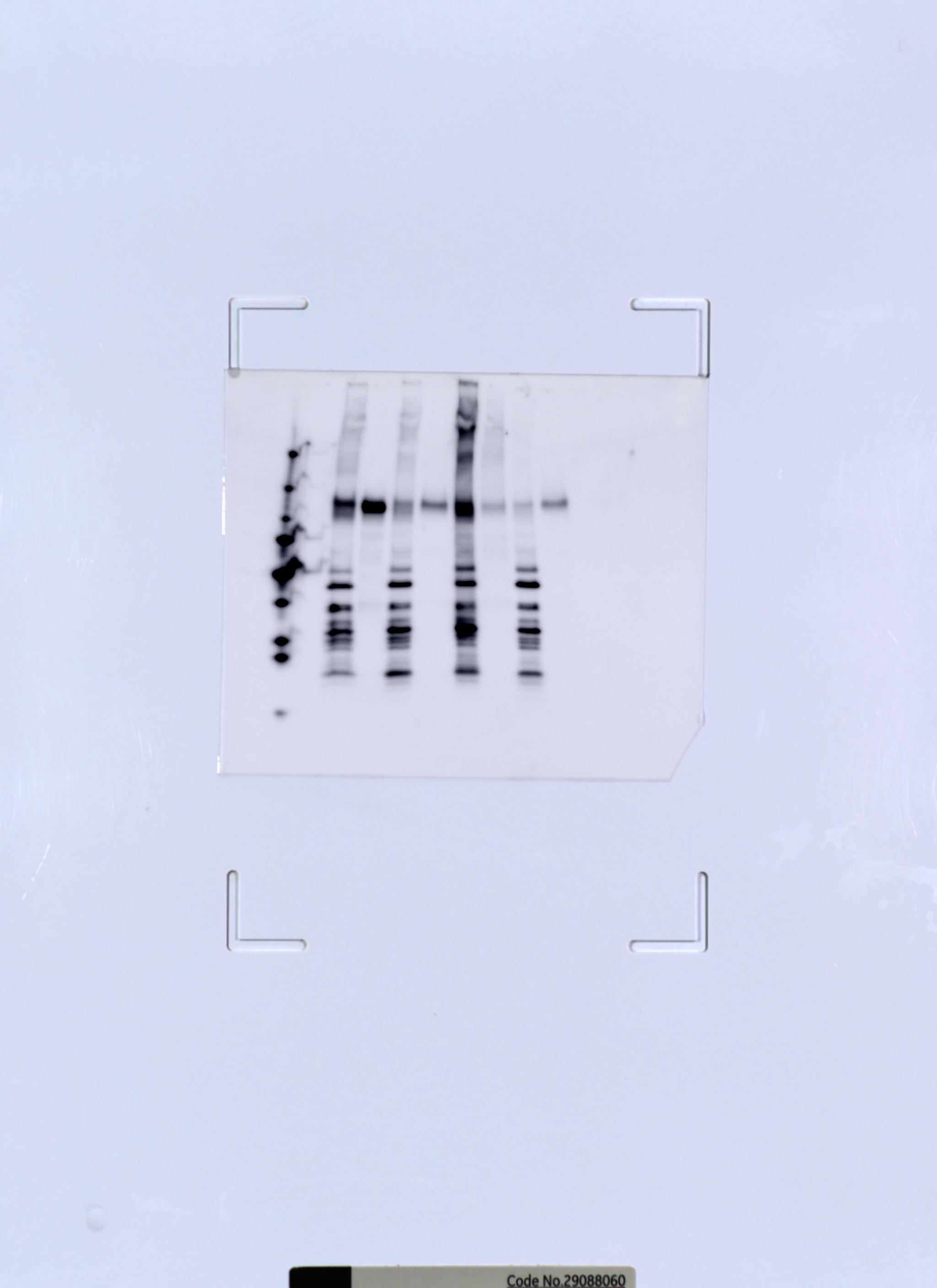

Supplement: Figure 2—figure supplement 4—source data 1. [file elife-100061-fig2-figsupp4-data1.zip › Figure 2ΓÇöfigure supplement 4ΓÇösource data 1/panel3_rep3_021924-bel-mutants-3c 2024.02.19_15.14.02_Ch+Marker.jpg]

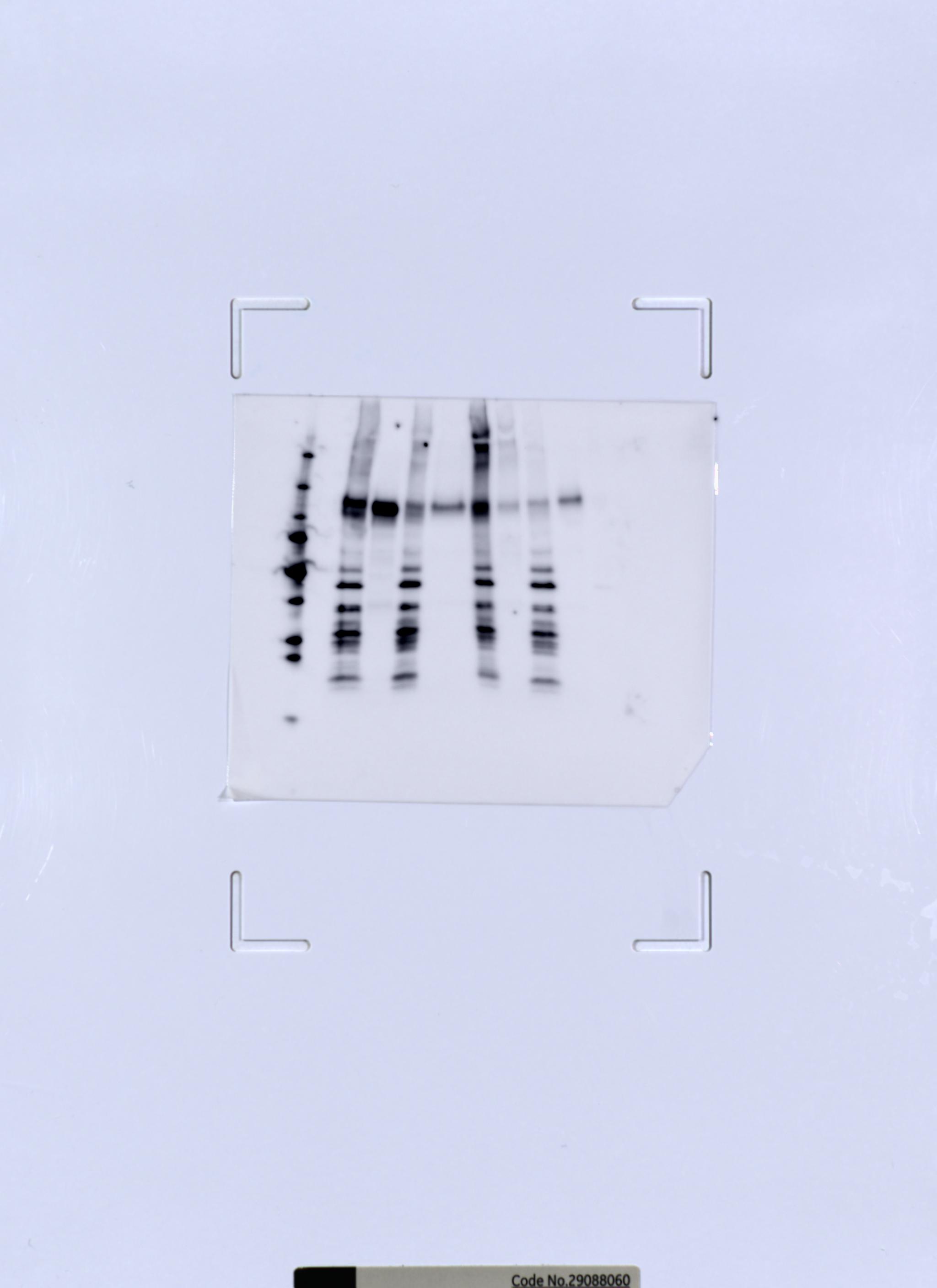

Supplement: Figure 2—figure supplement 4—source data 1. [file elife-100061-fig2-figsupp4-data1.zip › Figure 2ΓÇöfigure supplement 4ΓÇösource data 1/panel1_rep3_021924-bel-mutants-1C 2024.02.19_13.41.18_Ch+Marker.jpg]

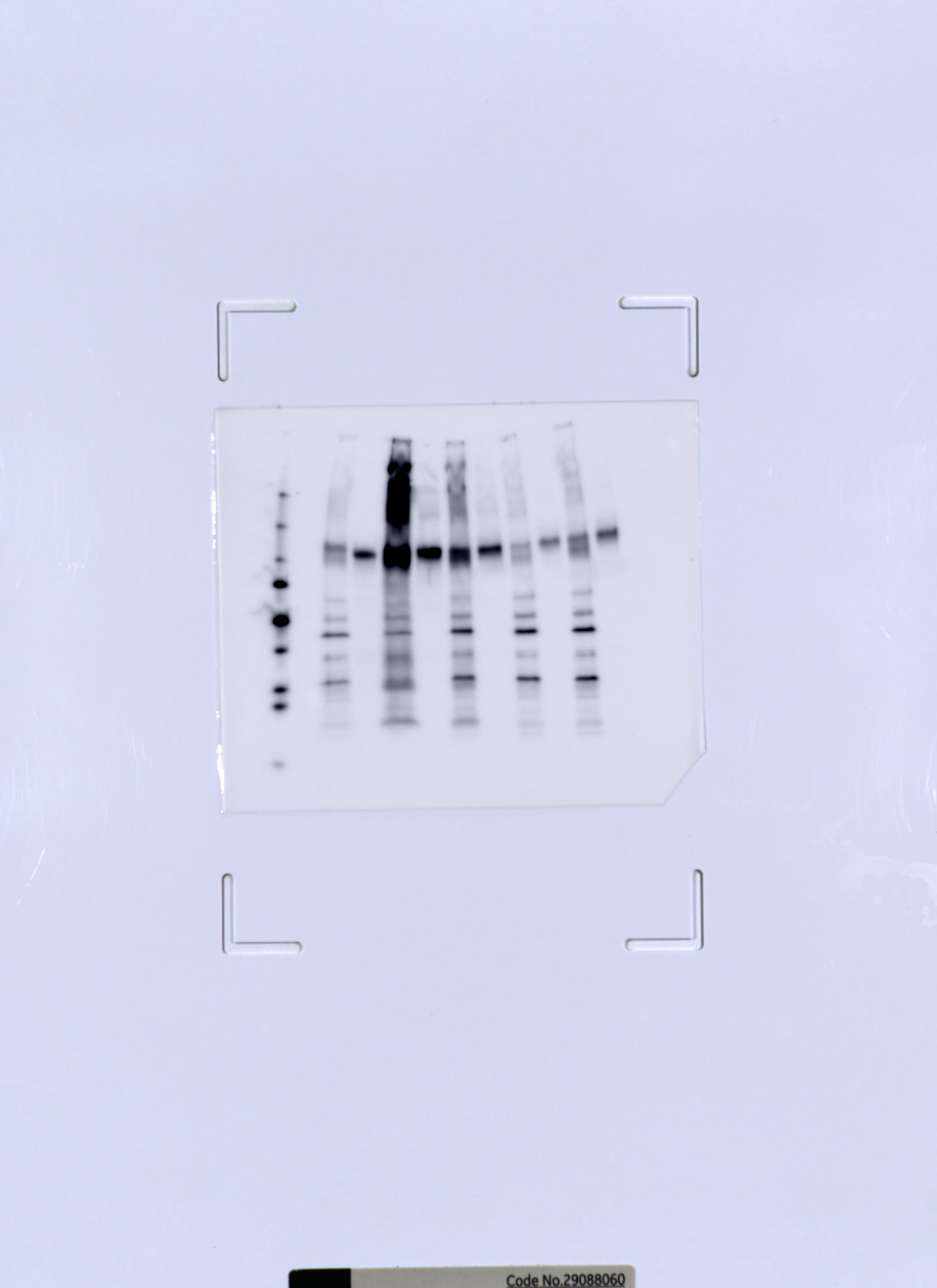

Supplement: Figure 2—figure supplement 4—source data 1. [file elife-100061-fig2-figsupp4-data1.zip › Figure 2ΓÇöfigure supplement 4ΓÇösource data 1/panel3_rep1_021924-bel-mutants-3A 2024.02.19_15.43.12_Ch+Marker.jpg]

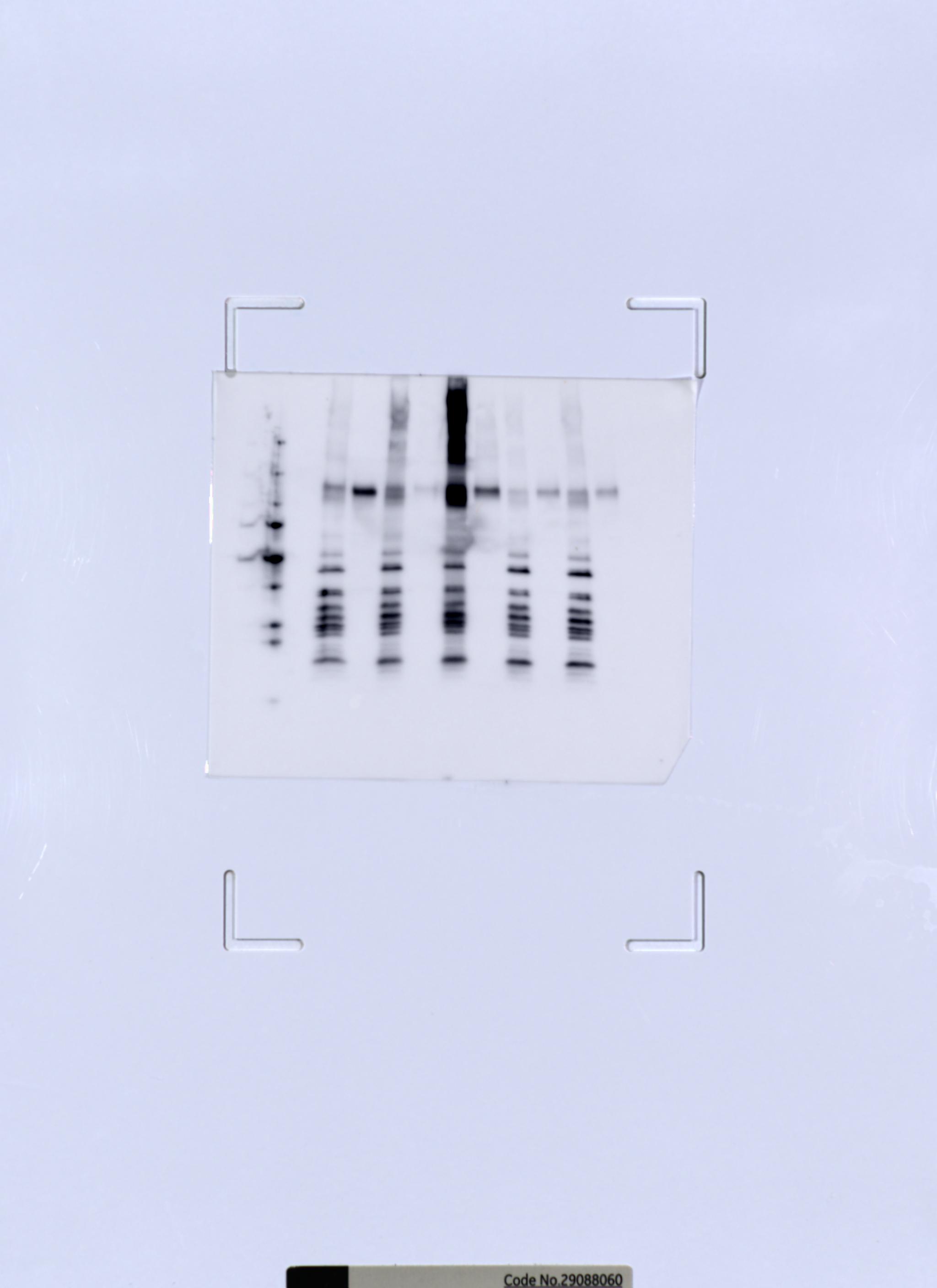

Supplement: Figure 2—figure supplement 4—source data 1. [file elife-100061-fig2-figsupp4-data1.zip › Figure 2ΓÇöfigure supplement 4ΓÇösource data 1/panel3_rep2_021924-bel-mutants-3B 2024.02.19_15.30.42_Ch+Marker.jpg]

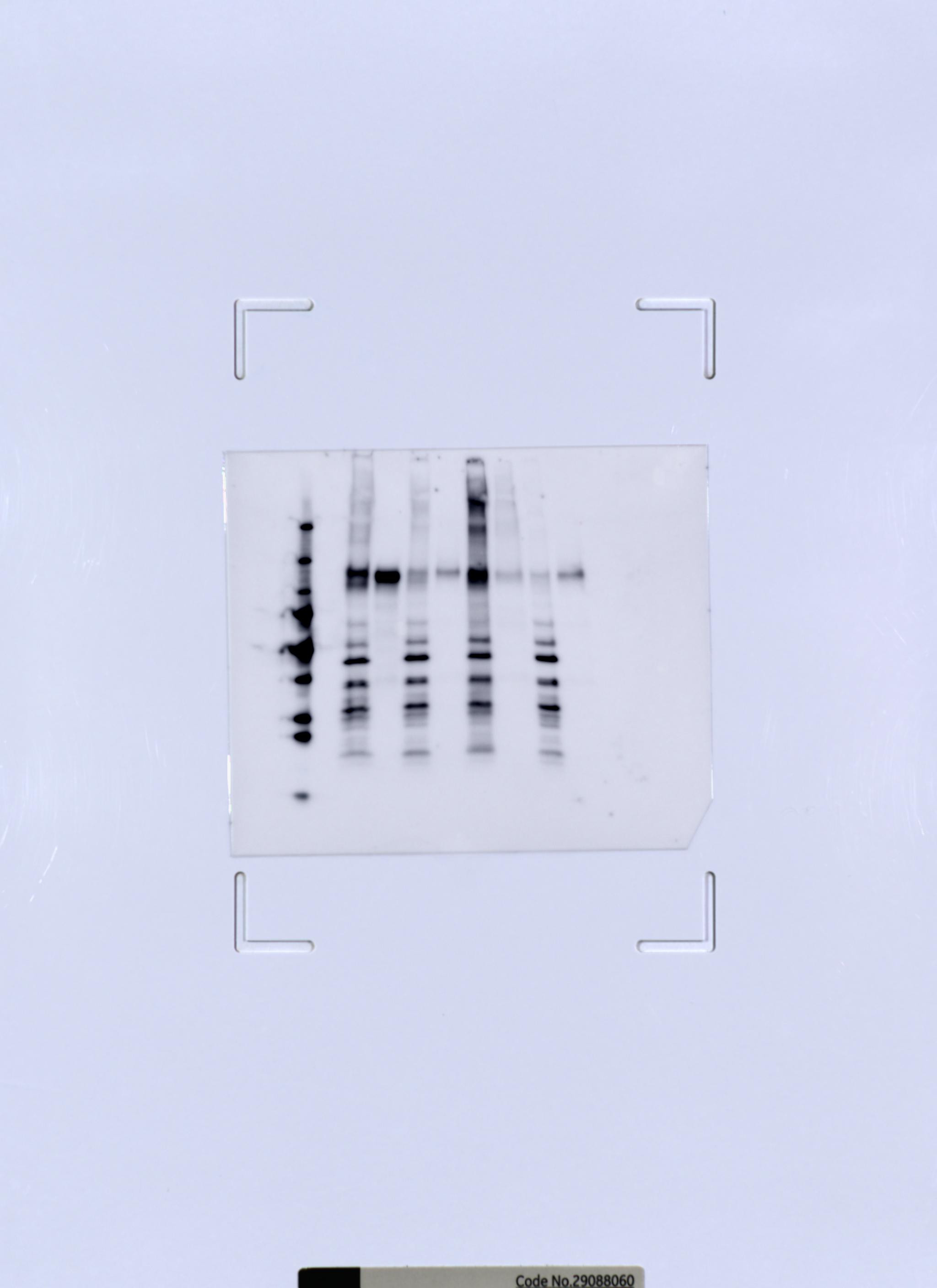

Supplement: Figure 2—figure supplement 4—source data 1. [file elife-100061-fig2-figsupp4-data1.zip › Figure 2ΓÇöfigure supplement 4ΓÇösource data 1/panel2_rep3_021924-bel-mutants-2C 2024.02.19_14.23.00_Ch+Marker.jpg]

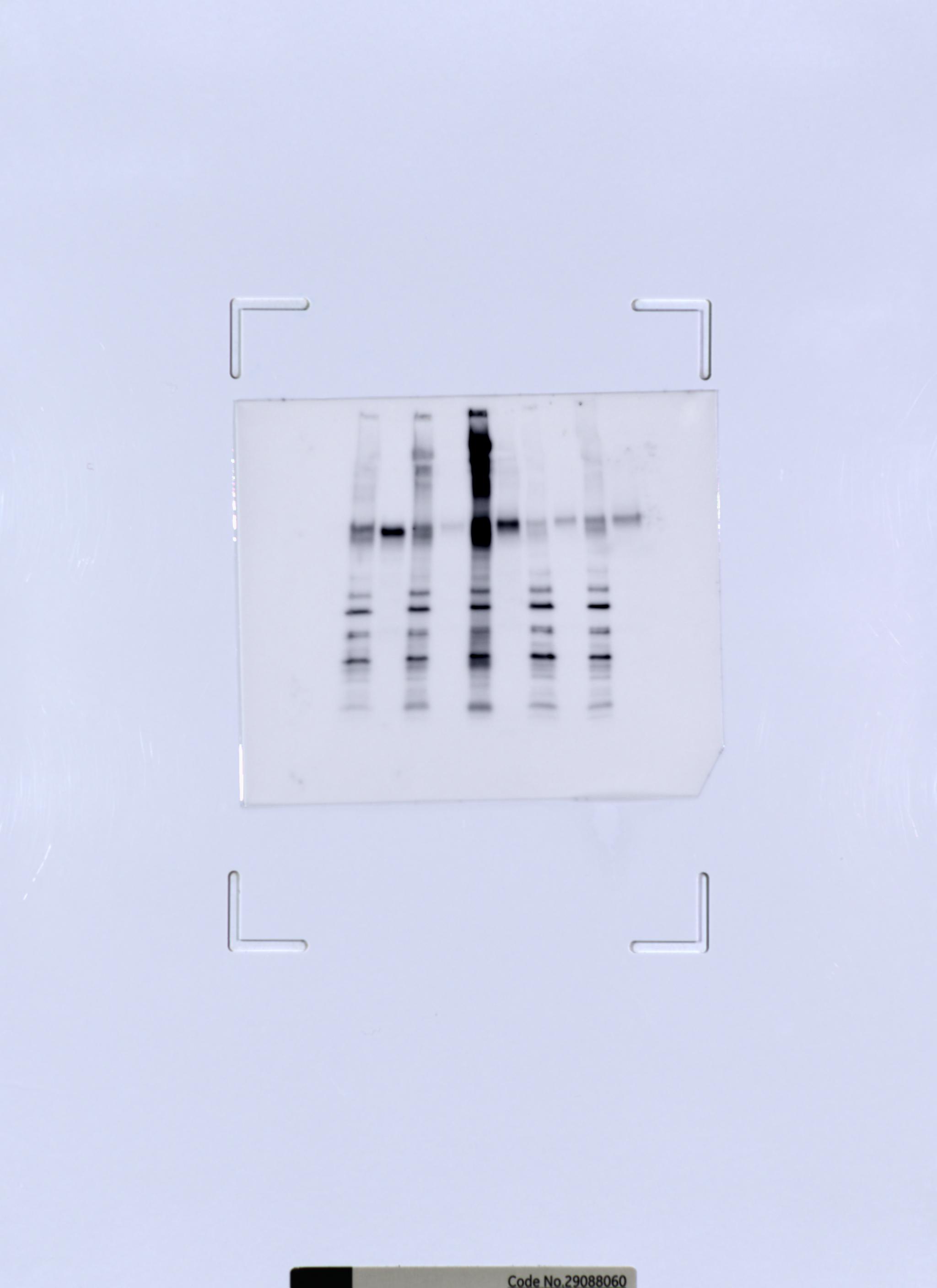

Supplement: Figure 2—figure supplement 4—source data 1. [file elife-100061-fig2-figsupp4-data1.zip › Figure 2ΓÇöfigure supplement 4ΓÇösource data 1/panel2_rep2_021924-bel-mutants-2B 2024.02.19_14.39.26_Ch+Marker.jpg]

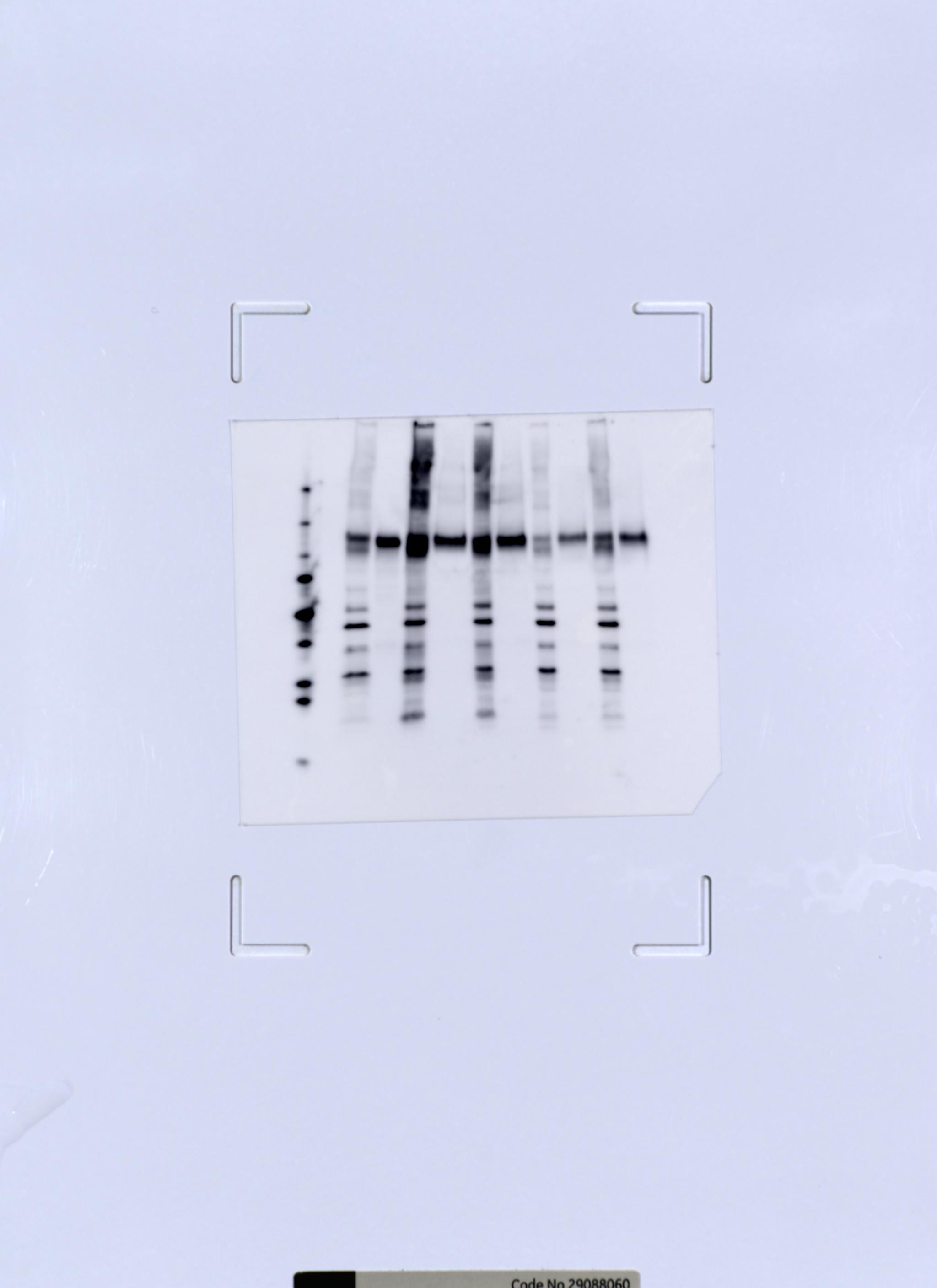

Supplement: Figure 2—figure supplement 4—source data 1. [file elife-100061-fig2-figsupp4-data1.zip › Figure 2ΓÇöfigure supplement 4ΓÇösource data 1/panel2_rep1_021924-bel-mutants-2A 2024.02.19_14.53.44_Ch+Marker.jpg]

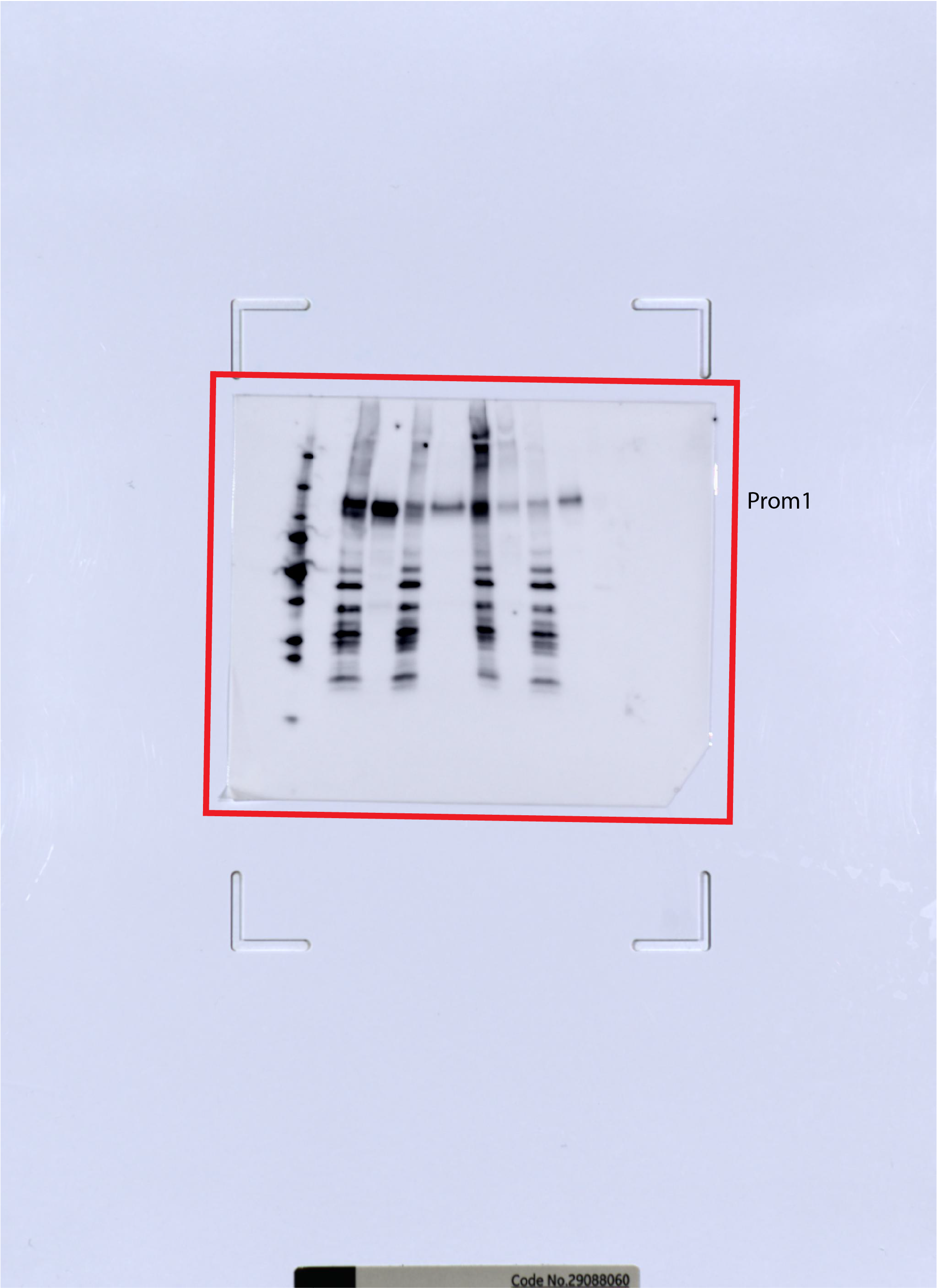

Supplement: Figure 2—figure supplement 4—source data 2. [file elife-100061-fig2-figsupp4-data2.zip › Figure 2ΓÇöfigure supplement 4ΓÇösource data 2/panel1_rep3_fig_WITH_RED_BOX_labeled.png]

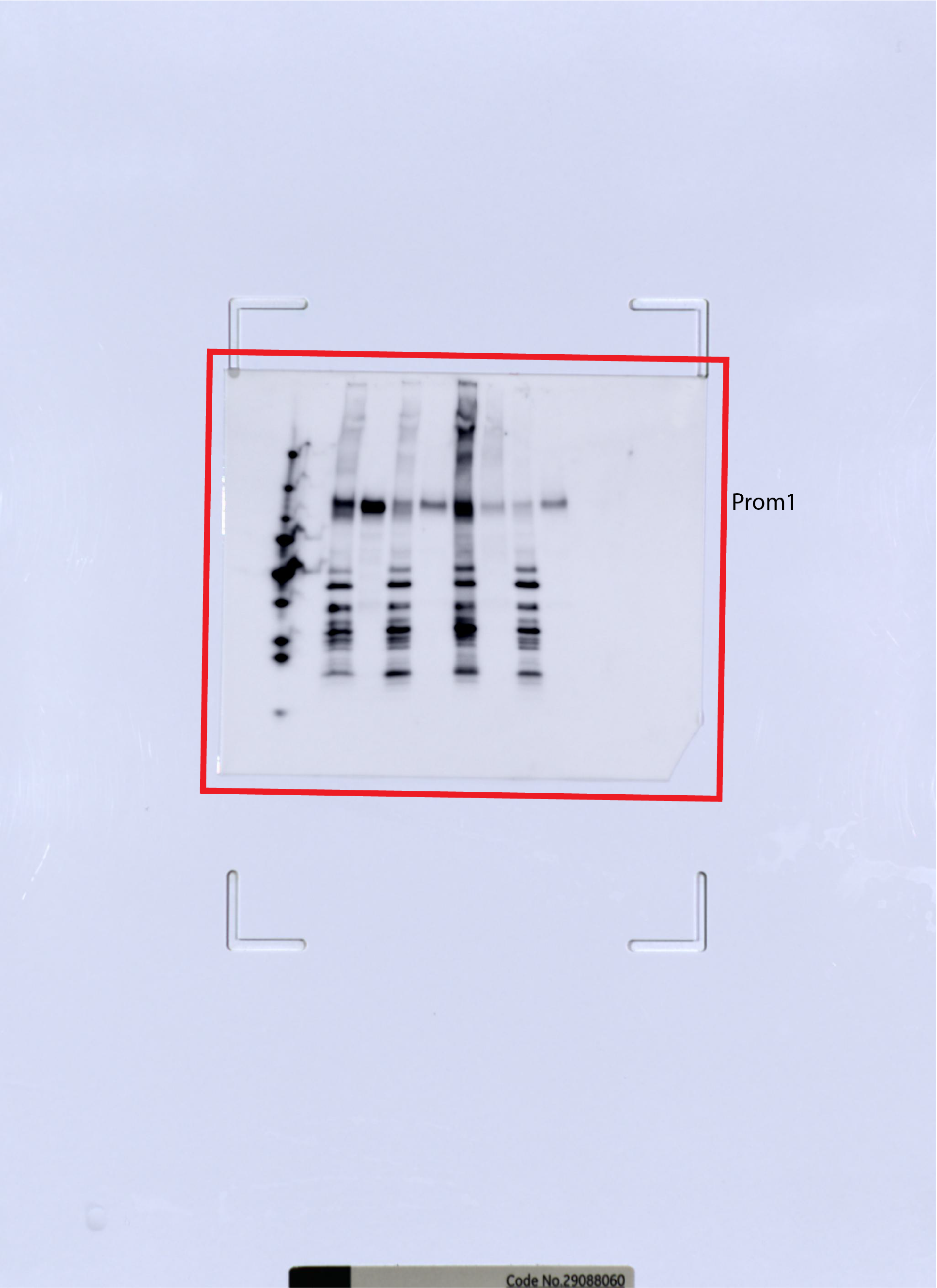

Supplement: Figure 2—figure supplement 4—source data 2. [file elife-100061-fig2-figsupp4-data2.zip › Figure 2ΓÇöfigure supplement 4ΓÇösource data 2/panel3_rep3_fig_WITH_RED_BOX_labeled.png]

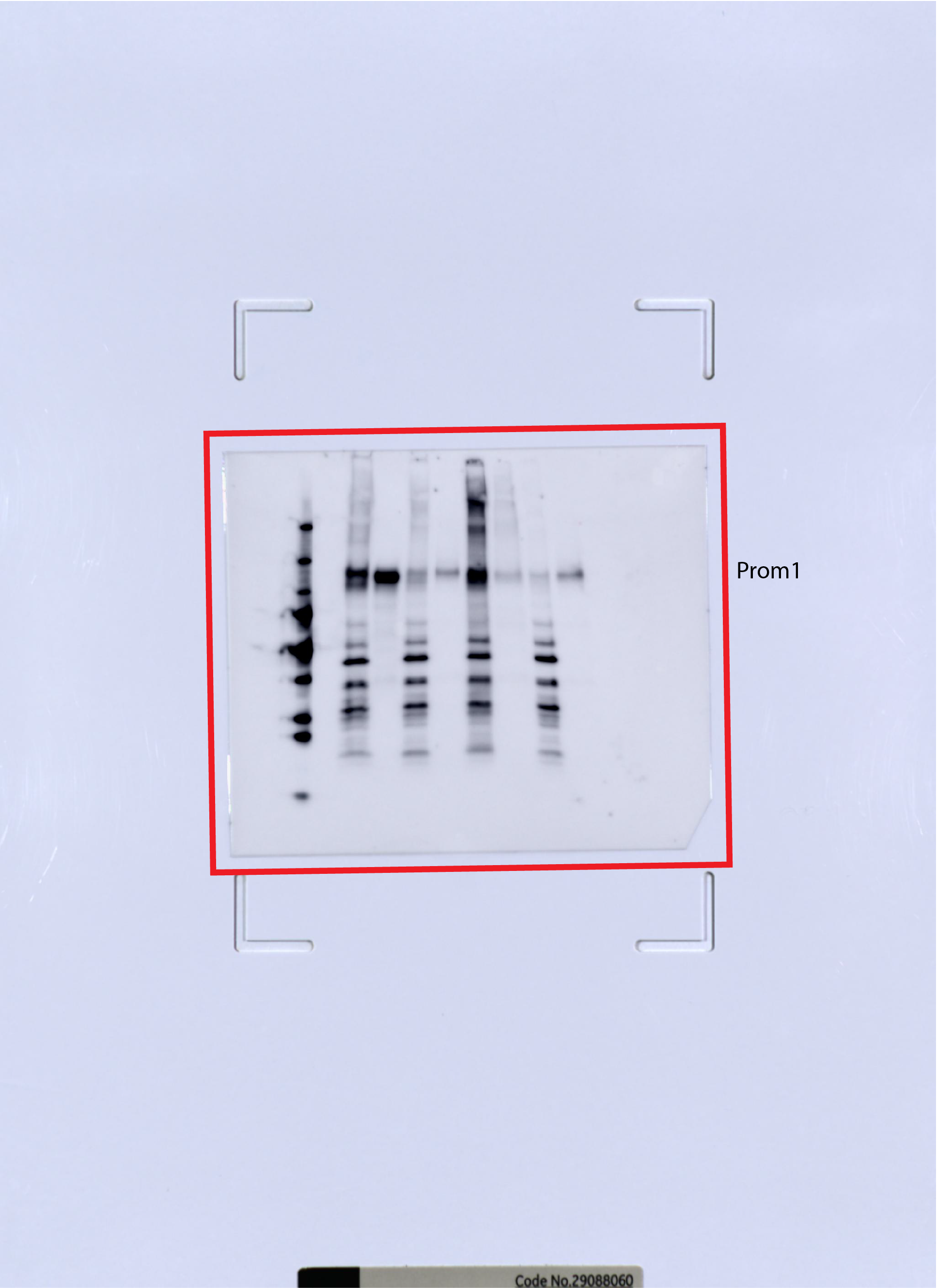

Supplement: Figure 2—figure supplement 4—source data 2. [file elife-100061-fig2-figsupp4-data2.zip › Figure 2ΓÇöfigure supplement 4ΓÇösource data 2/panel2_rep3_fig_WITH_RED_BOX_labeled.png]

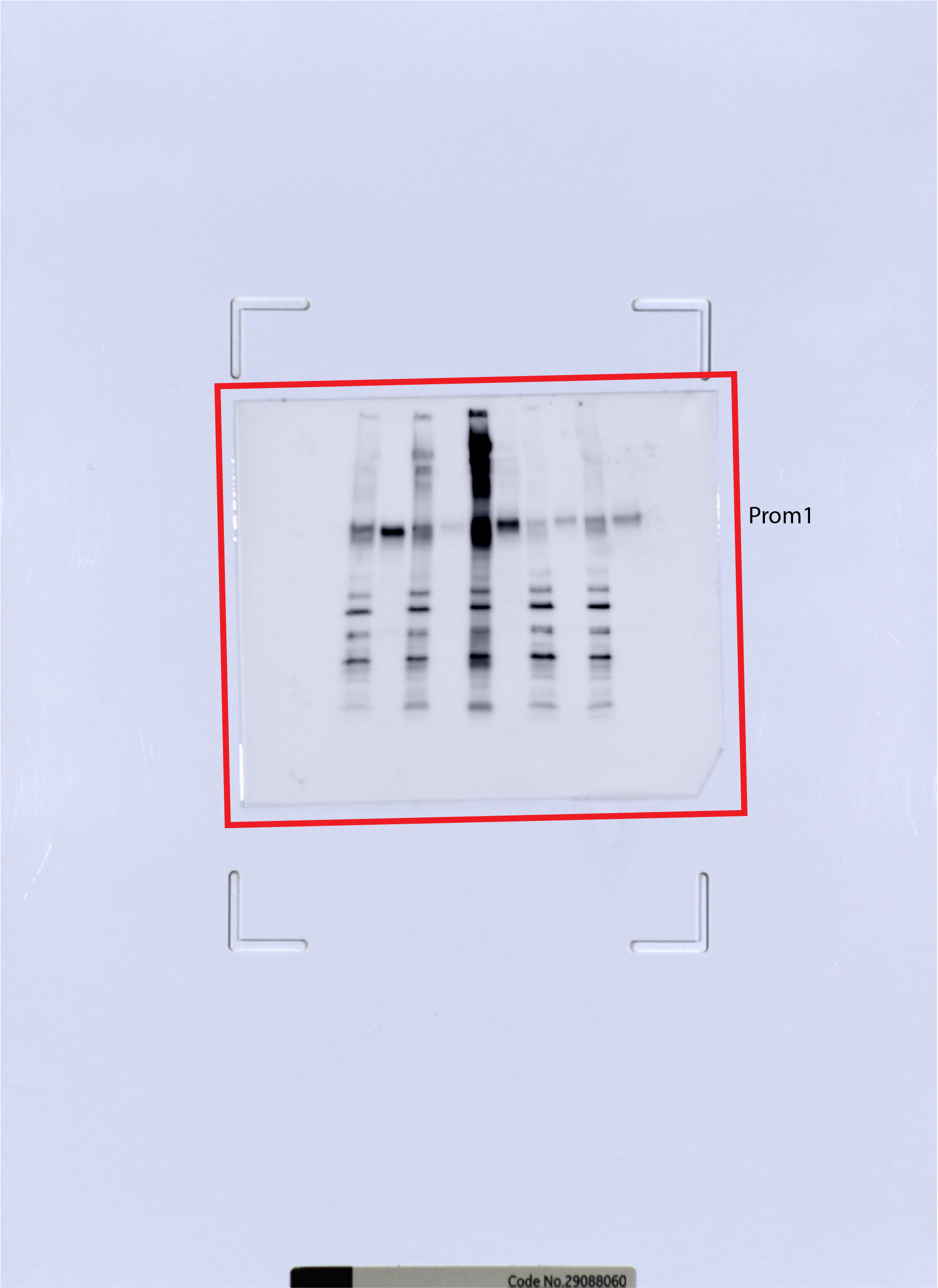

Supplement: Figure 2—figure supplement 4—source data 2. [file elife-100061-fig2-figsupp4-data2.zip › Figure 2ΓÇöfigure supplement 4ΓÇösource data 2/panel2_rep2_fig_WITH_RED_BOX_labeled.png]

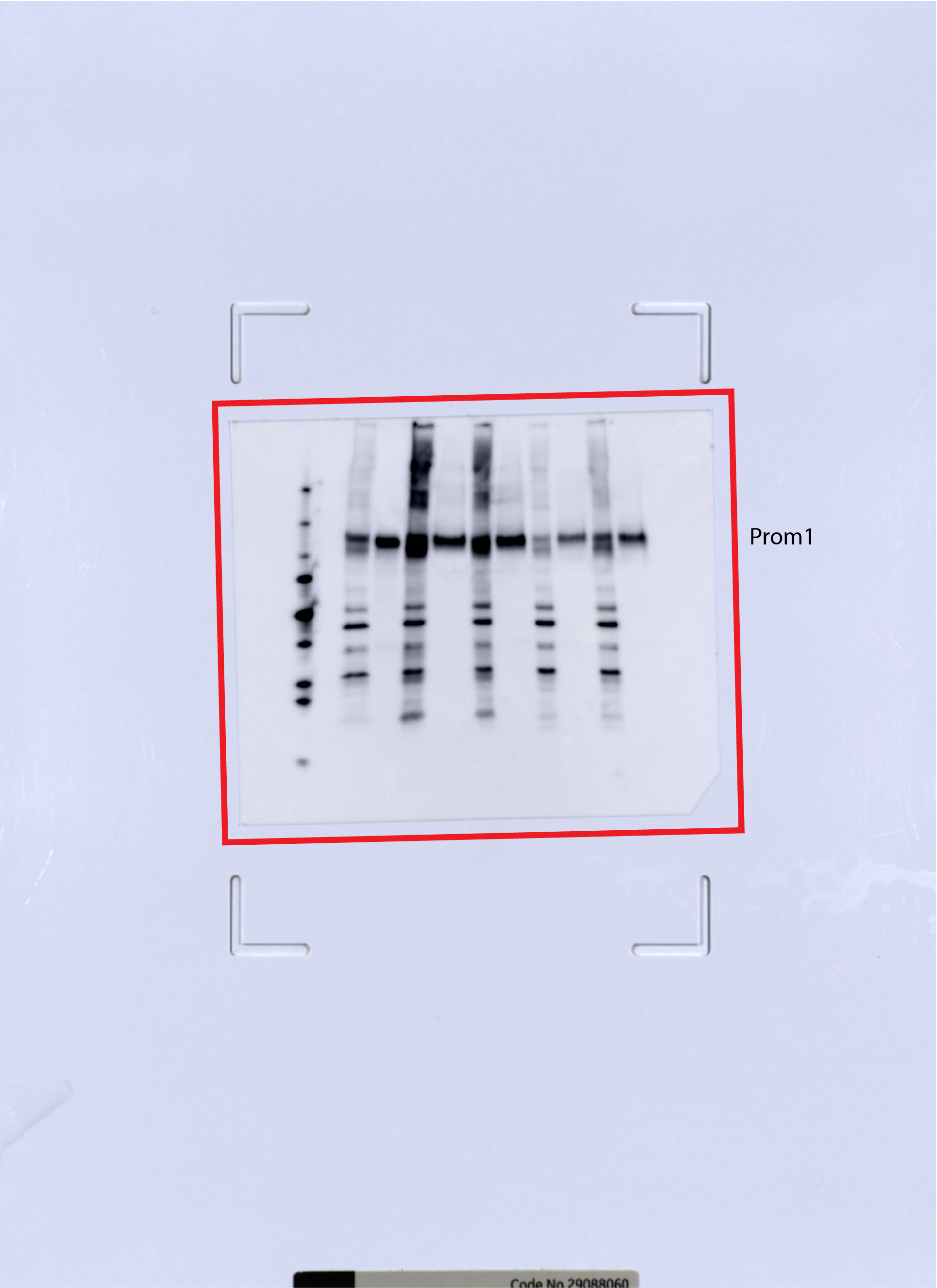

Supplement: Figure 2—figure supplement 4—source data 2. [file elife-100061-fig2-figsupp4-data2.zip › Figure 2ΓÇöfigure supplement 4ΓÇösource data 2/panel2_rep1_fig_WITH_RED_BOX_labeled.png]

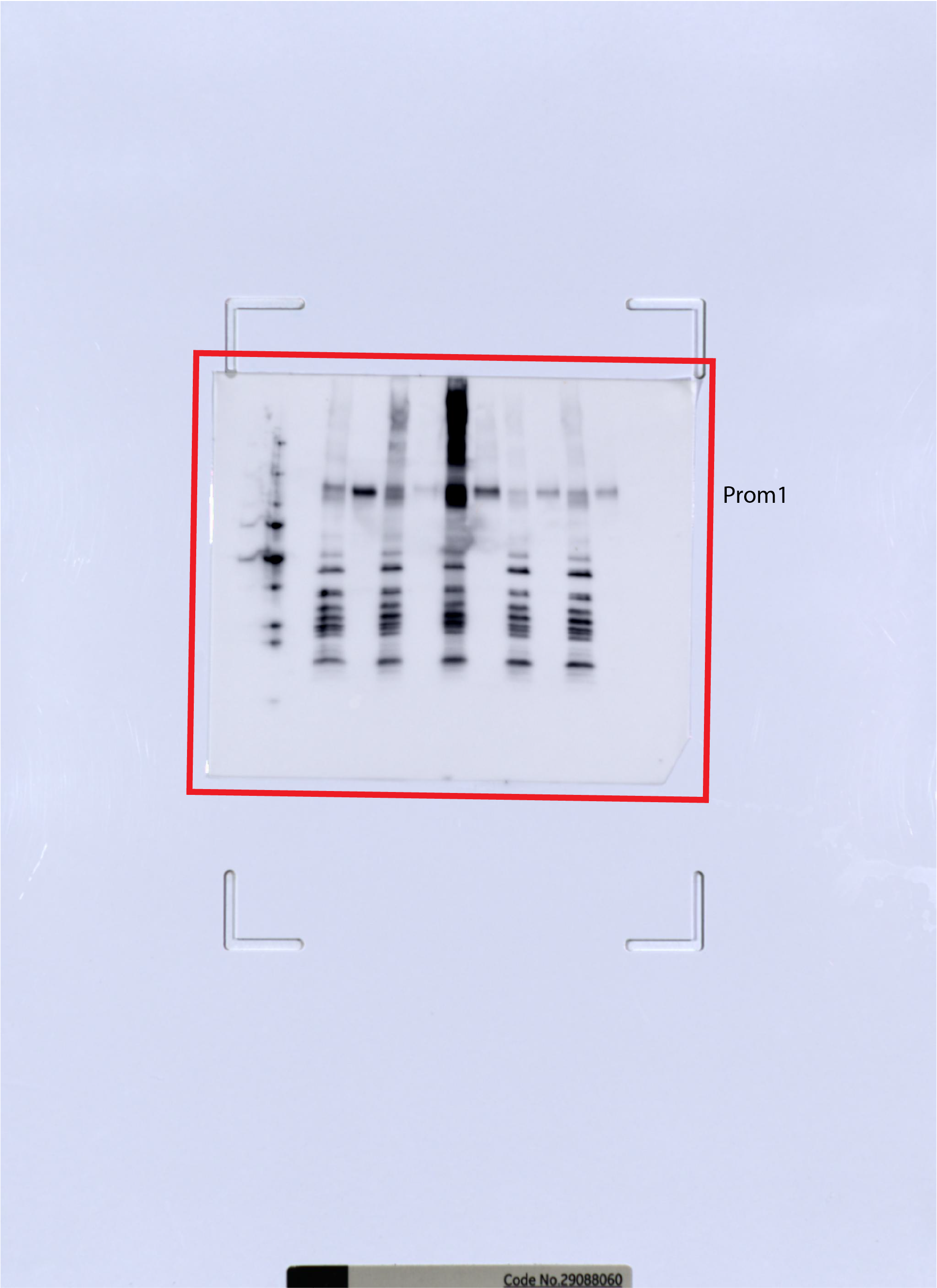

Supplement: Figure 2—figure supplement 4—source data 2. [file elife-100061-fig2-figsupp4-data2.zip › Figure 2ΓÇöfigure supplement 4ΓÇösource data 2/panel3_rep2_fig_WITH_RED_BOX_labeled.png]

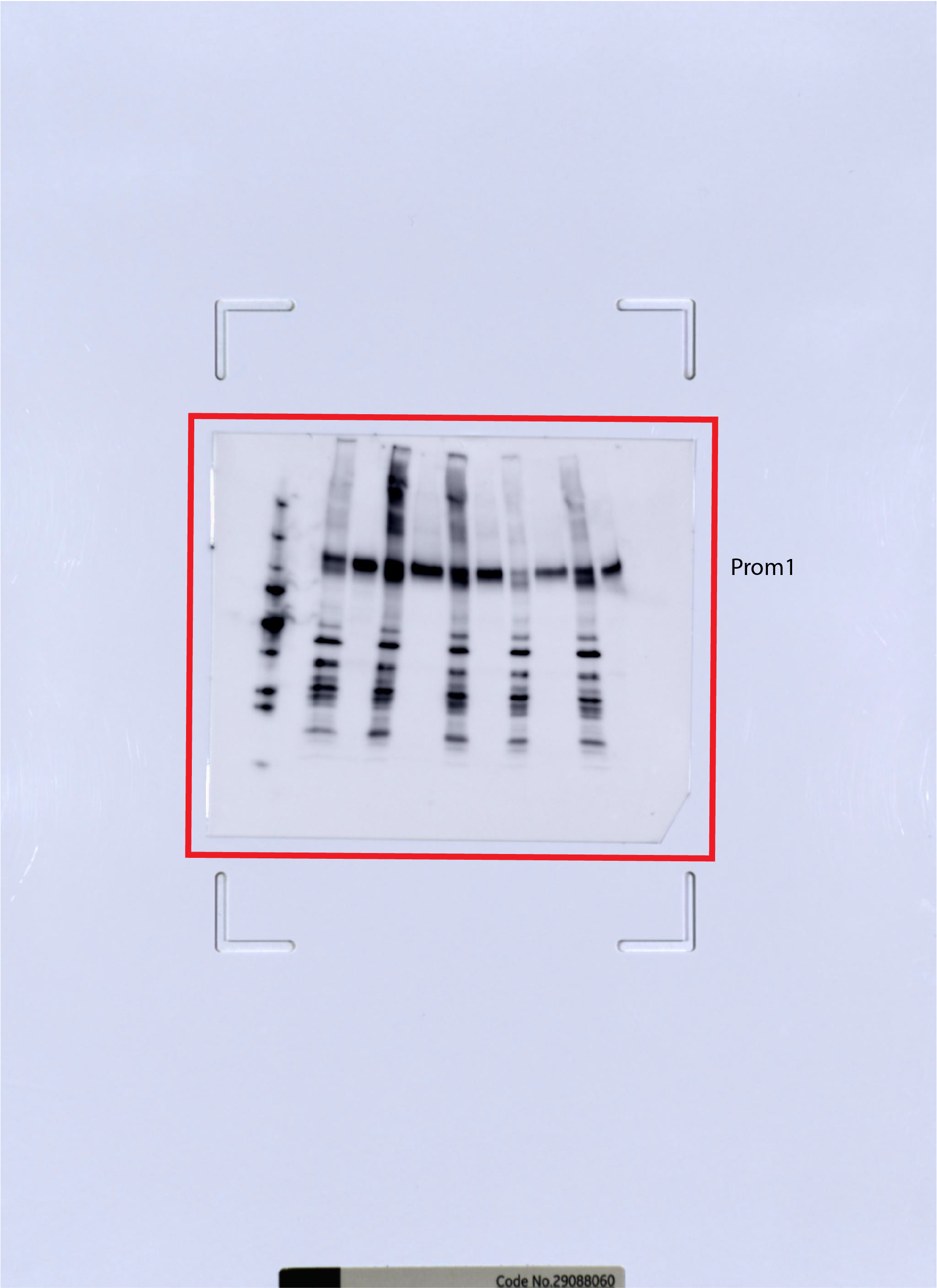

Supplement: Figure 2—figure supplement 4—source data 2. [file elife-100061-fig2-figsupp4-data2.zip › Figure 2ΓÇöfigure supplement 4ΓÇösource data 2/panel1_rep1_fig_WITH_RED_BOX_labeled.png]

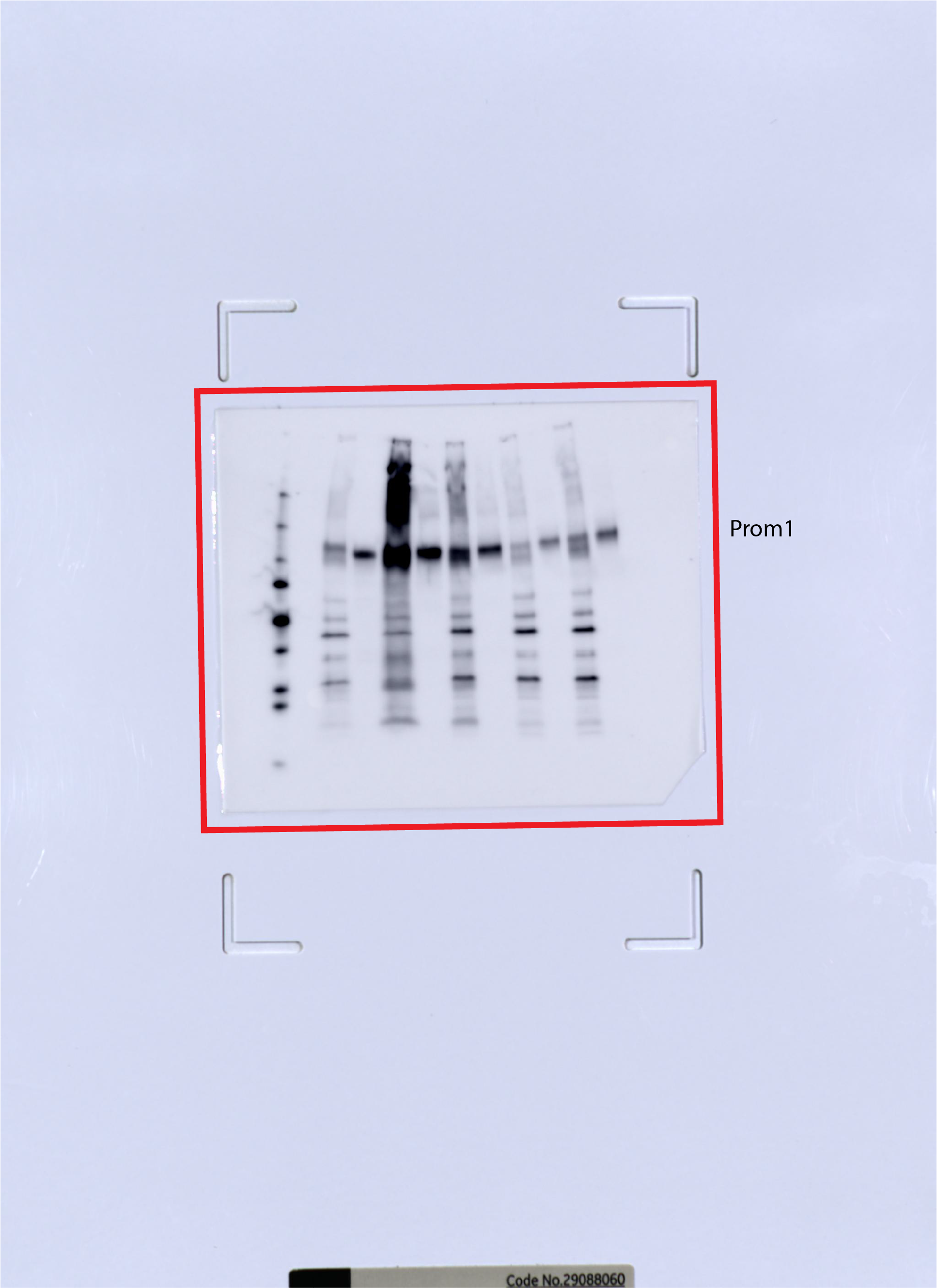

Supplement: Figure 2—figure supplement 4—source data 2. [file elife-100061-fig2-figsupp4-data2.zip › Figure 2ΓÇöfigure supplement 4ΓÇösource data 2/panel3_rep1_fig_WITH_RED_BOX_labeled.png]

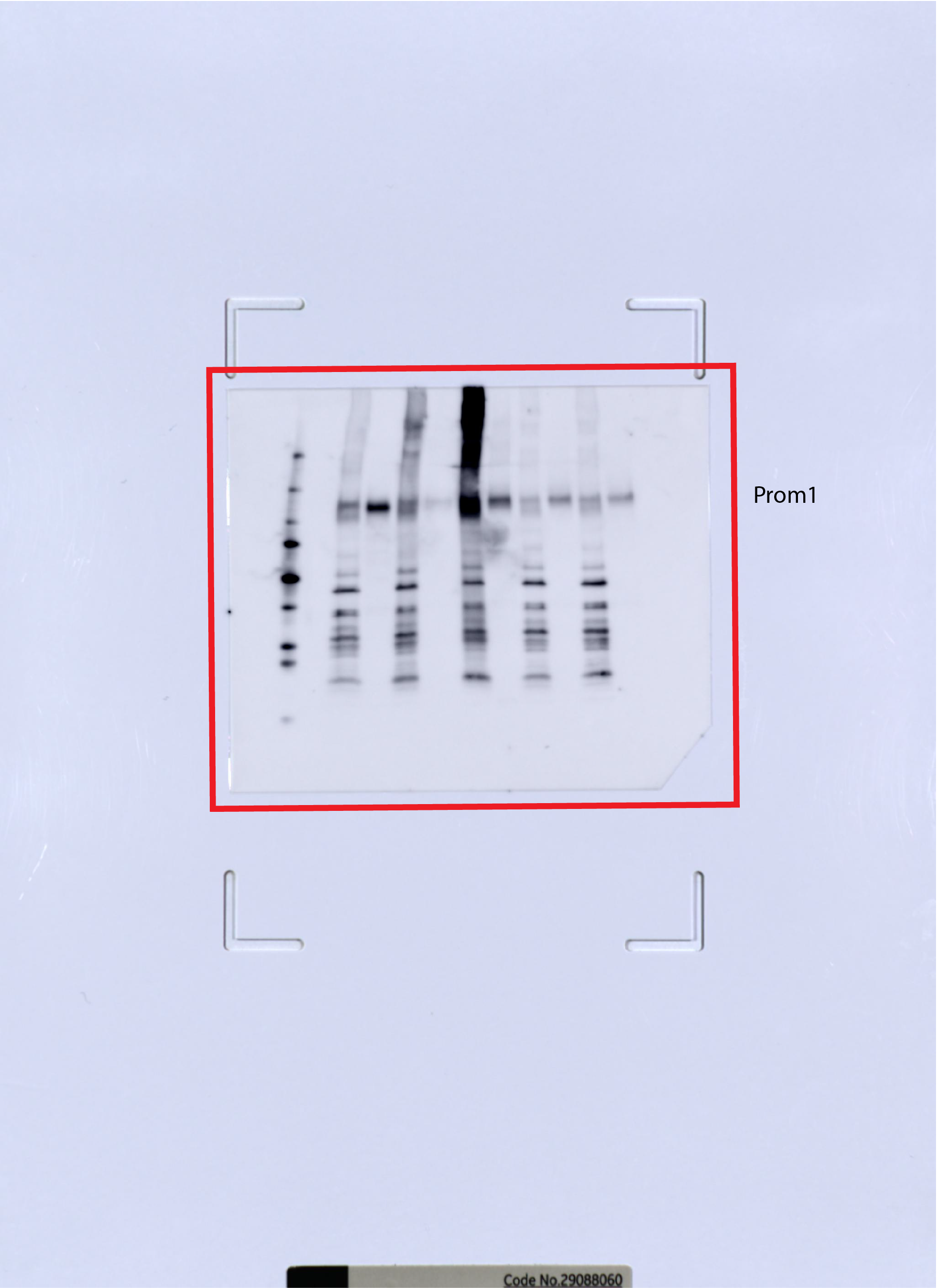

Supplement: Figure 2—figure supplement 4—source data 2. [file elife-100061-fig2-figsupp4-data2.zip › Figure 2ΓÇöfigure supplement 4ΓÇösource data 2/panel1_rep2_fig_WITH_RED_BOX_labeled.png]

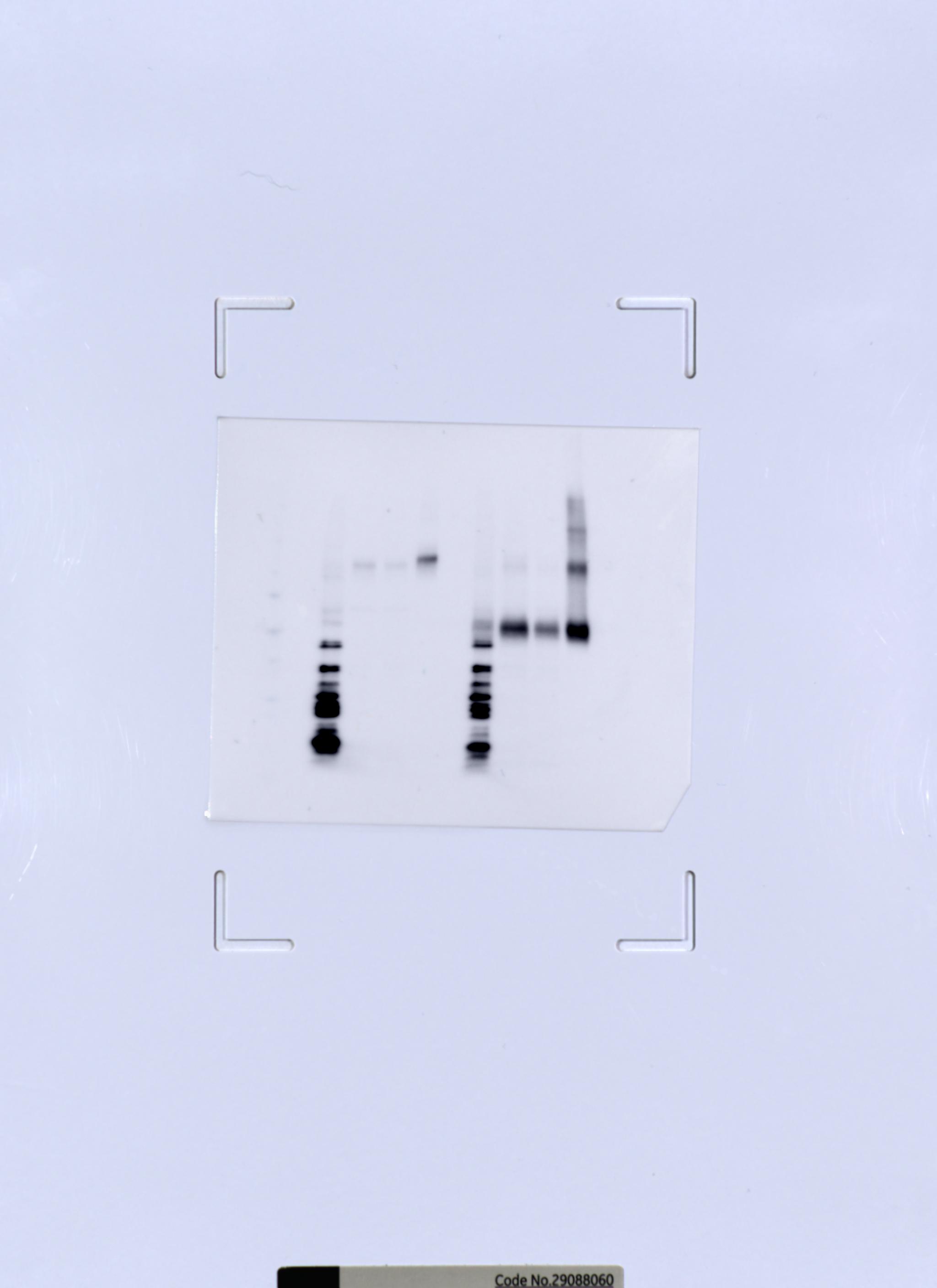

Supplement: Figure 3—source data 1. [file elife-100061-fig3-data1.zip › Figure 3-source data 1/Fig3C_022224-promvttyh 2024.02.22_13.24.44_Ch+Marker.jpg]

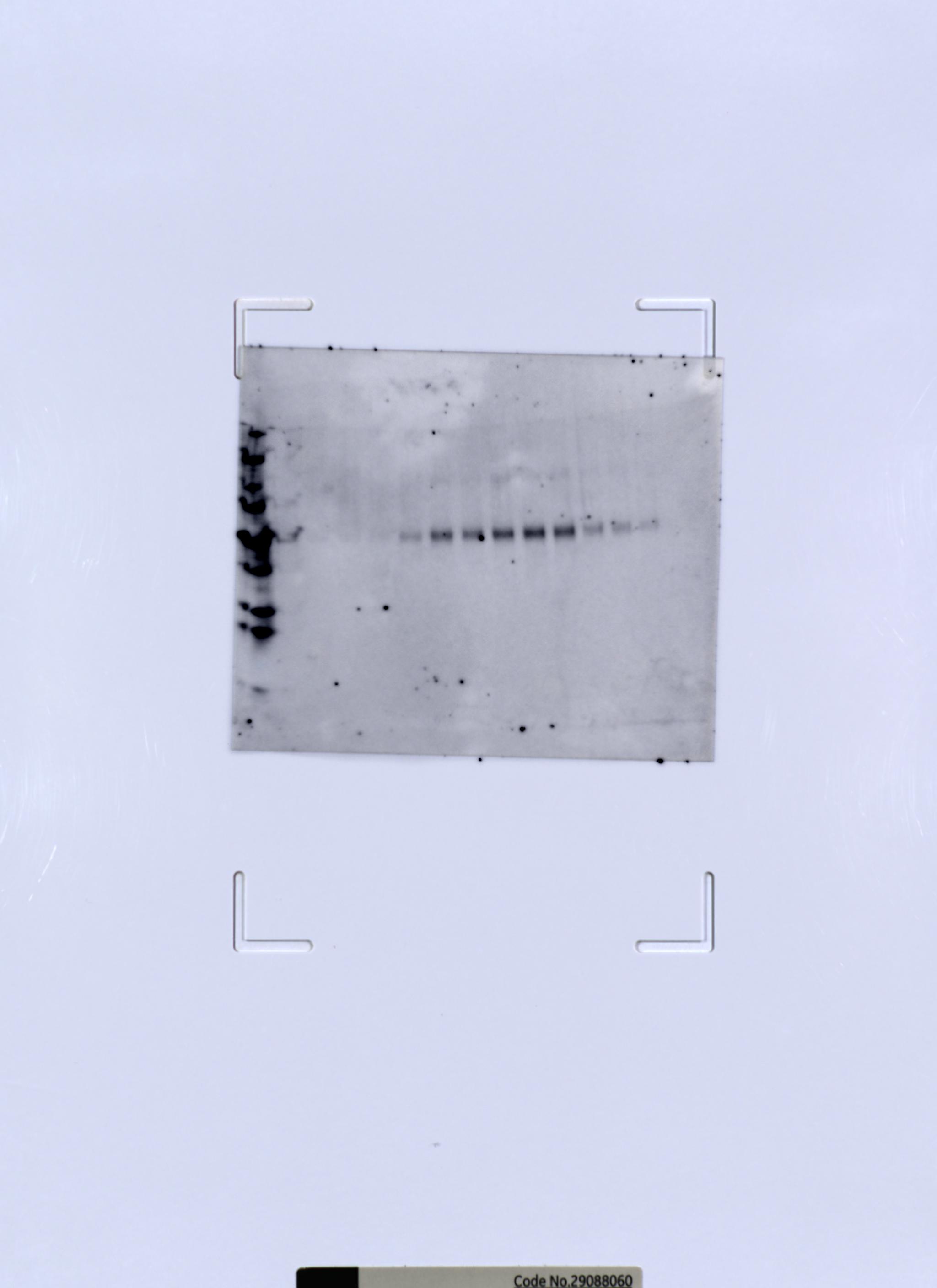

Supplement: Figure 3—source data 1. [file elife-100061-fig3-data1.zip › Figure 3-source data 1/Fig3J_bottom_230630-ttyh-a 2023.06.30_12.41.02_Ch+Marker.jpg]

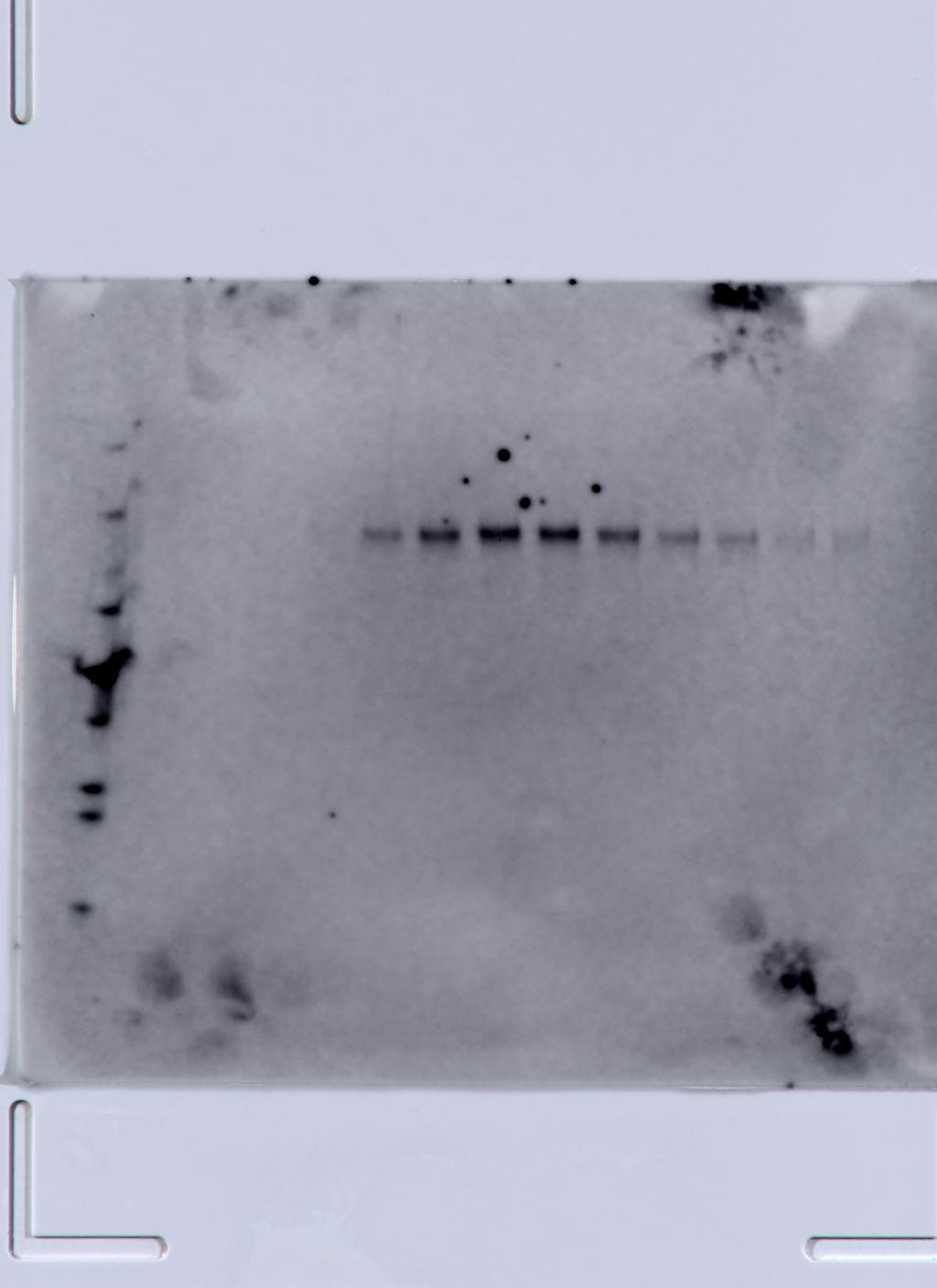

Supplement: Figure 3—source data 1. [file elife-100061-fig3-data1.zip › Figure 3-source data 1/Fig3J_top_wt prom1-b bel 230816 2023.08.16_16.14.52_Ch+Marker.jpg]

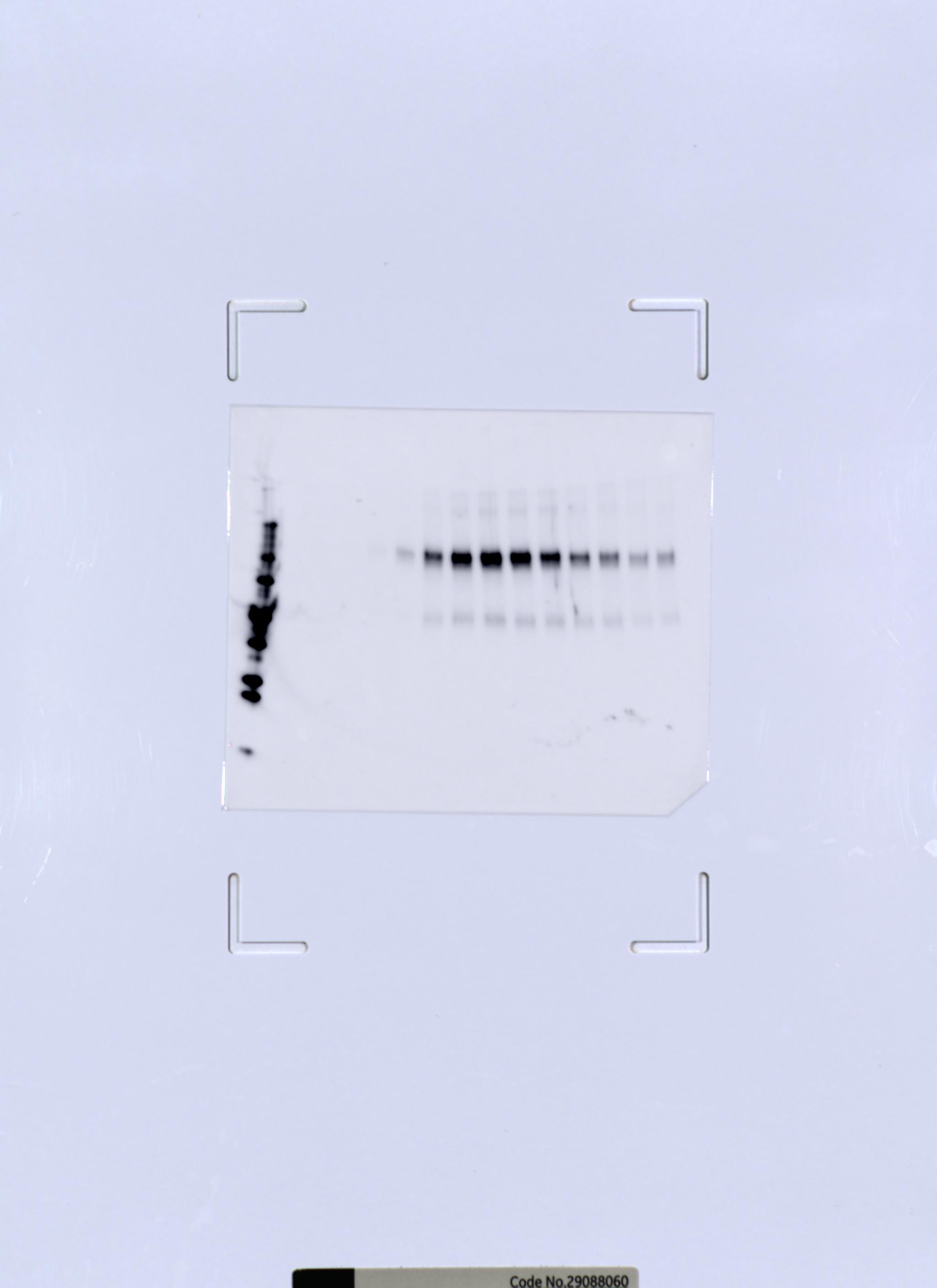

Supplement: Figure 3—source data 1. [file elife-100061-fig3-data1.zip › Figure 3-source data 1/Fig3K_20230921-promttyh c 2023.09.21_15.01.18_Ch+Marker.jpg]

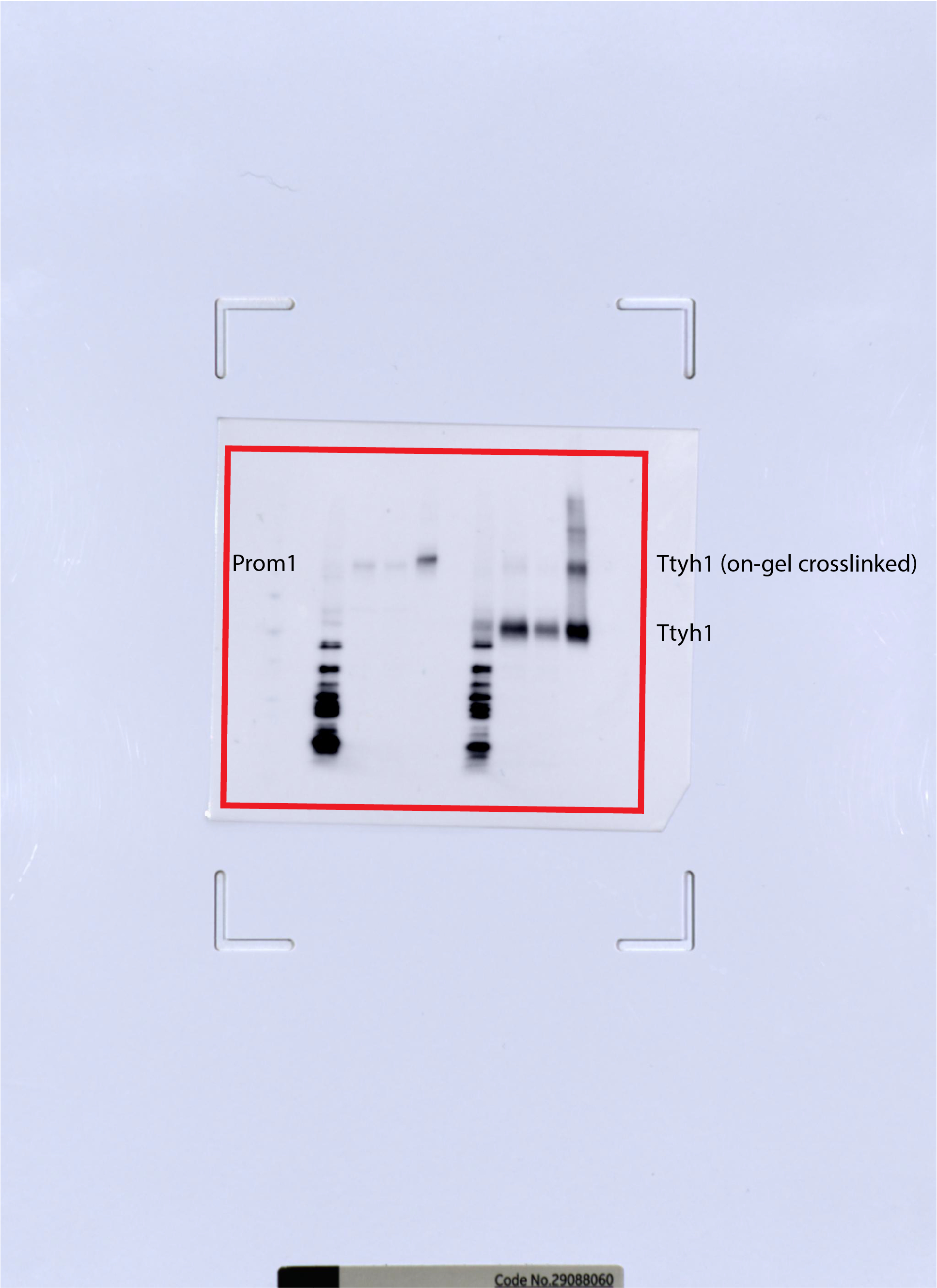

Supplement: Figure 3—source data 2. [file elife-100061-fig3-data2.zip › Figure 3-source data 2/Fig3C_WITH_RED_BOX_labeled.png]

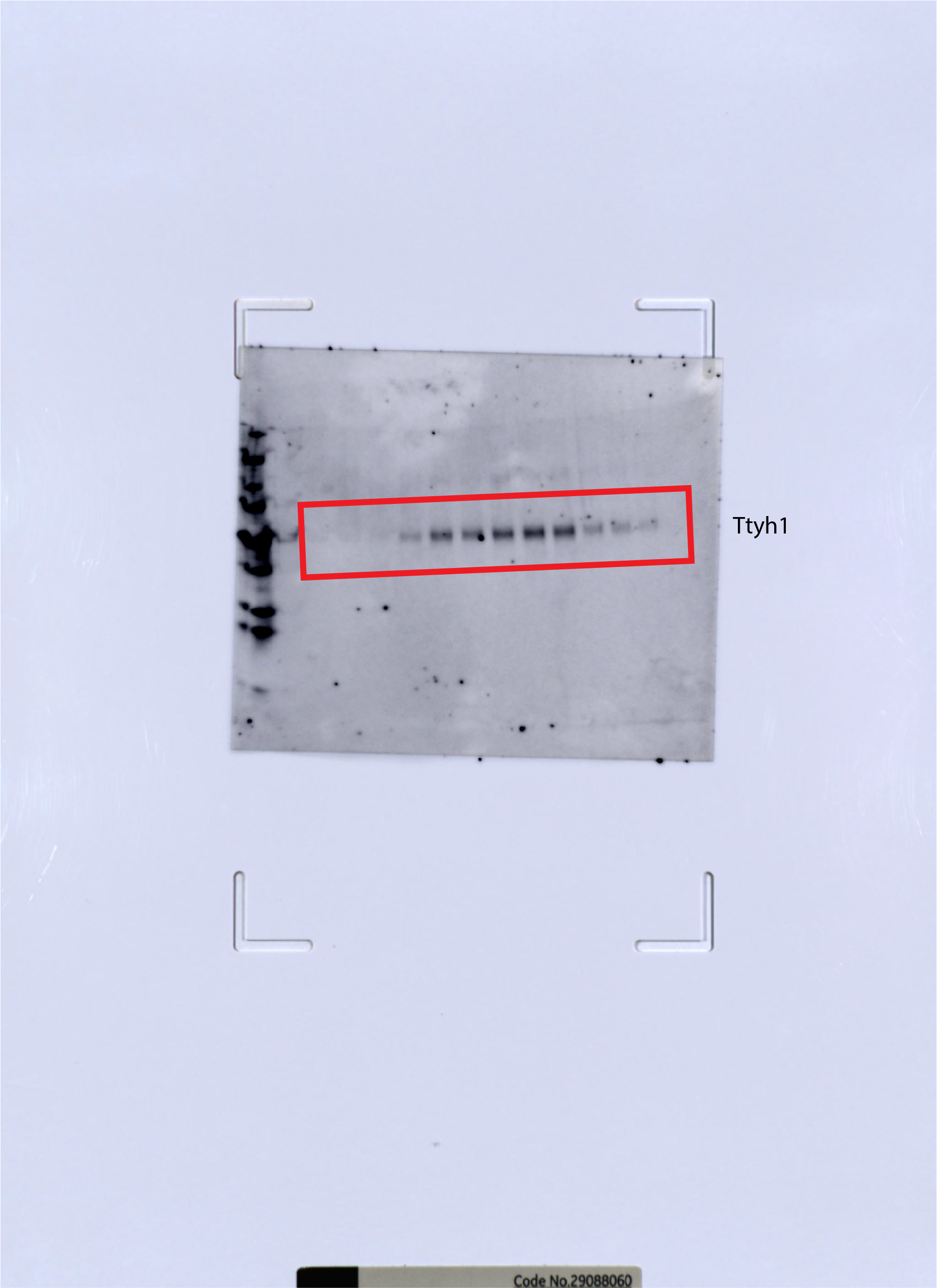

Supplement: Figure 3—source data 2. [file elife-100061-fig3-data2.zip › Figure 3-source data 2/Fig3J_bottom_WITH_RED_BOX_labeled.png]

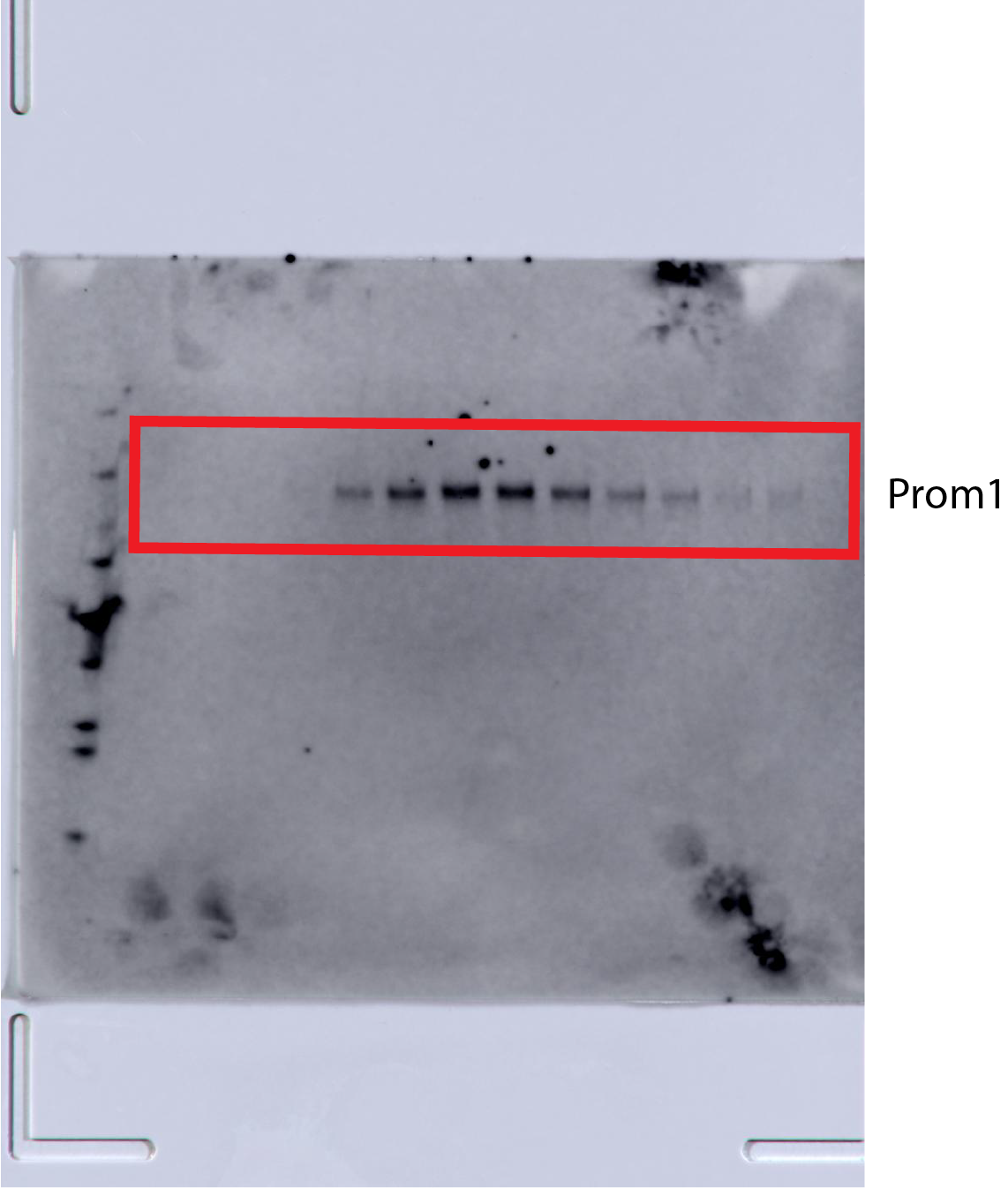

Supplement: Figure 3—source data 2. [file elife-100061-fig3-data2.zip › Figure 3-source data 2/Fig3J_top_WITH_RED_BOX_labeled.png]

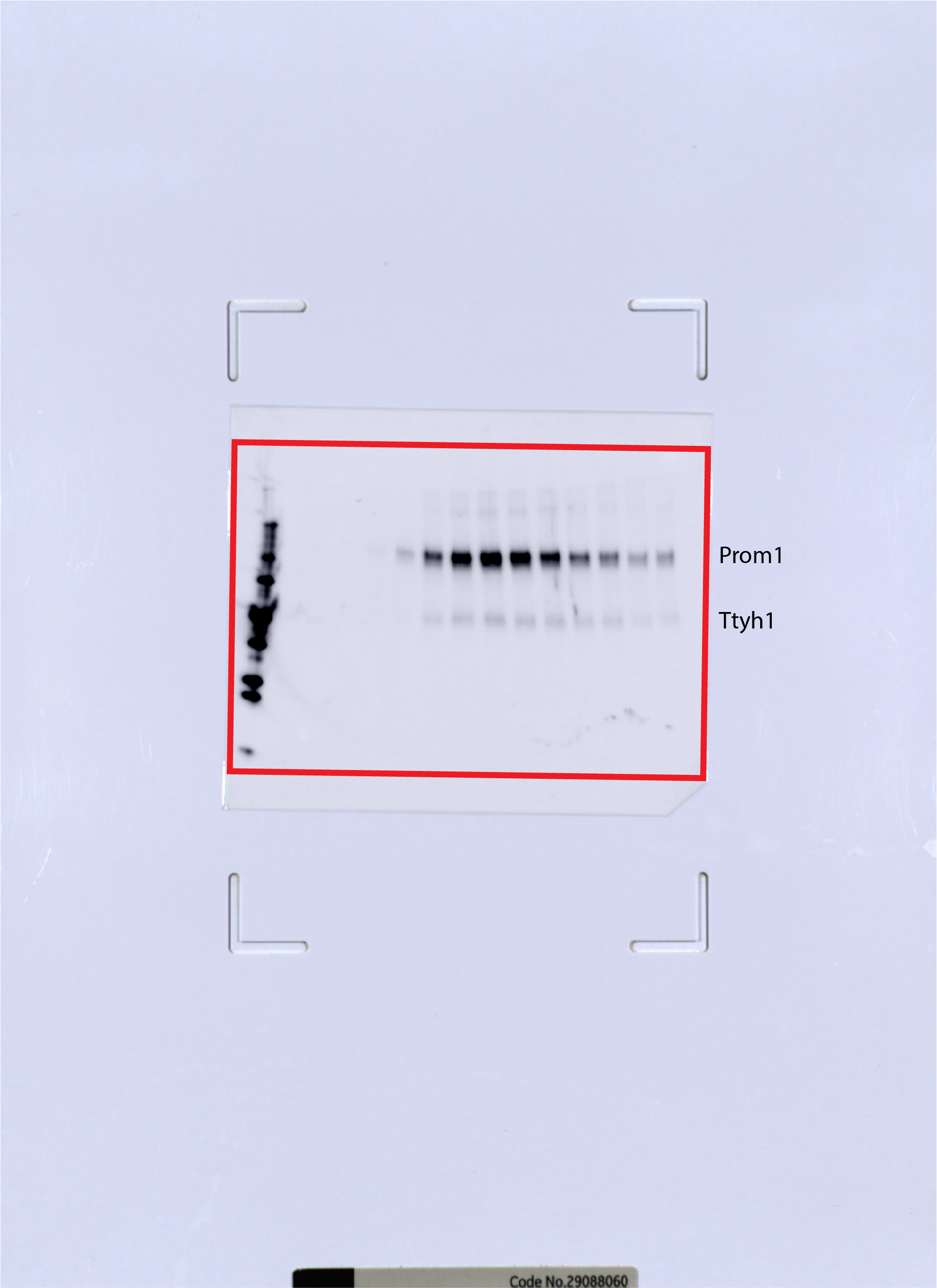

Supplement: Figure 3—source data 2. [file elife-100061-fig3-data2.zip › Figure 3-source data 2/Fig3K_WITH_RED_BOX_labeled.png]

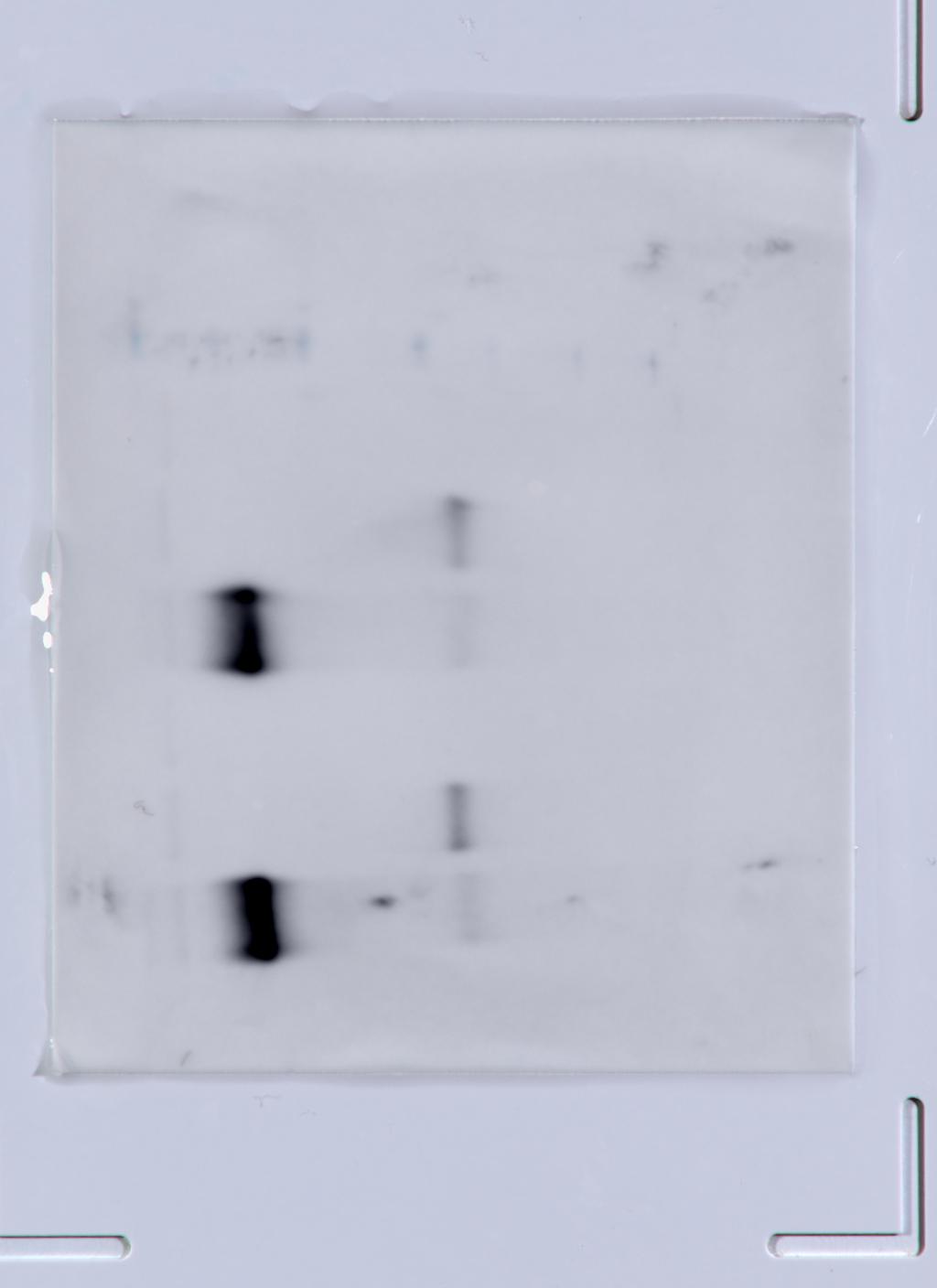

Supplement: Figure 3—figure supplement 1—source data 1. [file elife-100061-fig3-figsupp1-data1.zip › Figure 3-figure supplement 1-source data 1/Fig1B_left_240214-norm2 2024.02.14_11.32.39_Ch+Marker.jpg]

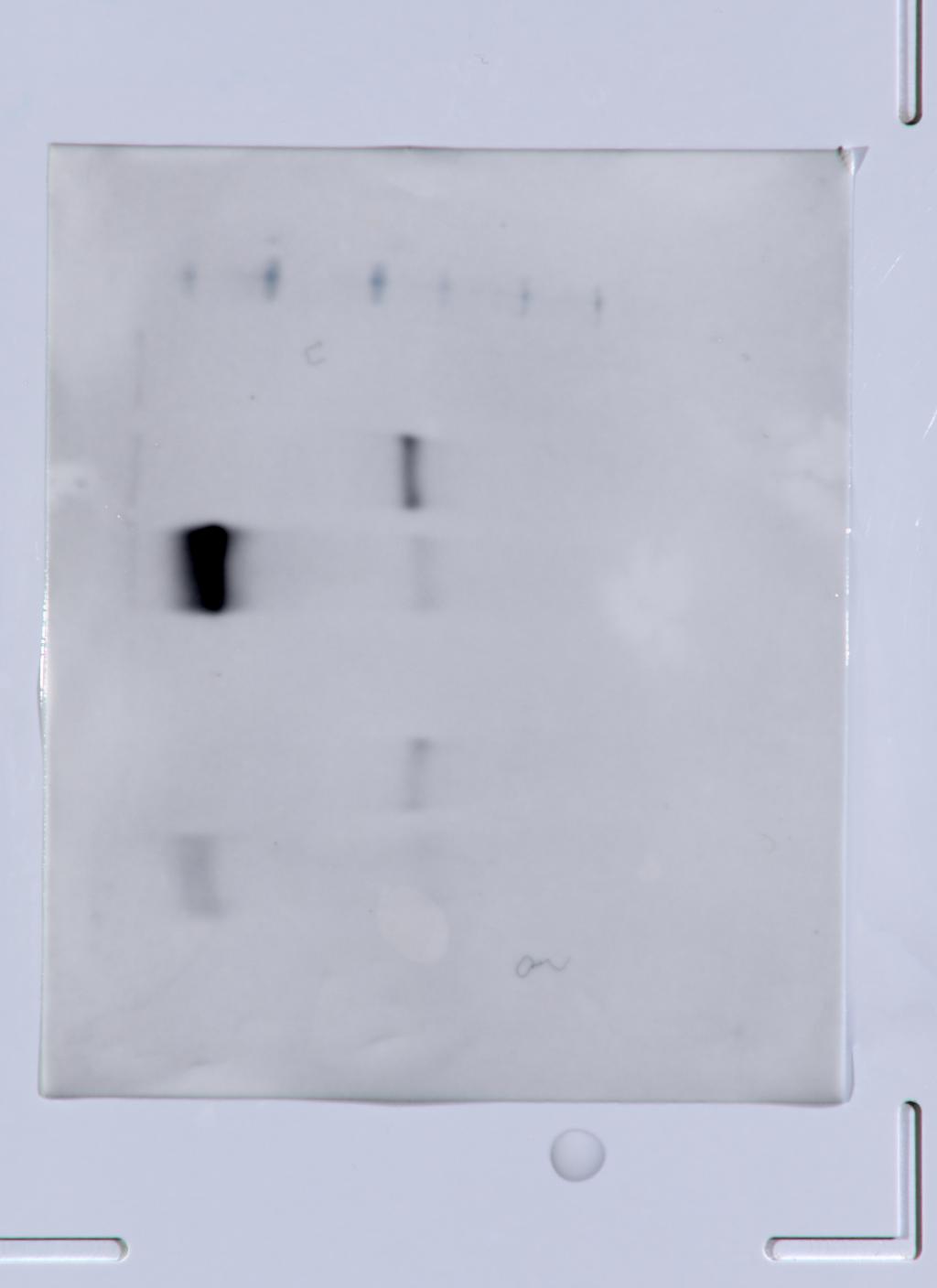

Supplement: Figure 3—figure supplement 1—source data 1. [file elife-100061-fig3-figsupp1-data1.zip › Figure 3-figure supplement 1-source data 1/Fig1B_right_240214-norm1 2024.02.14_11.21.07_Ch+Marker.jpg]

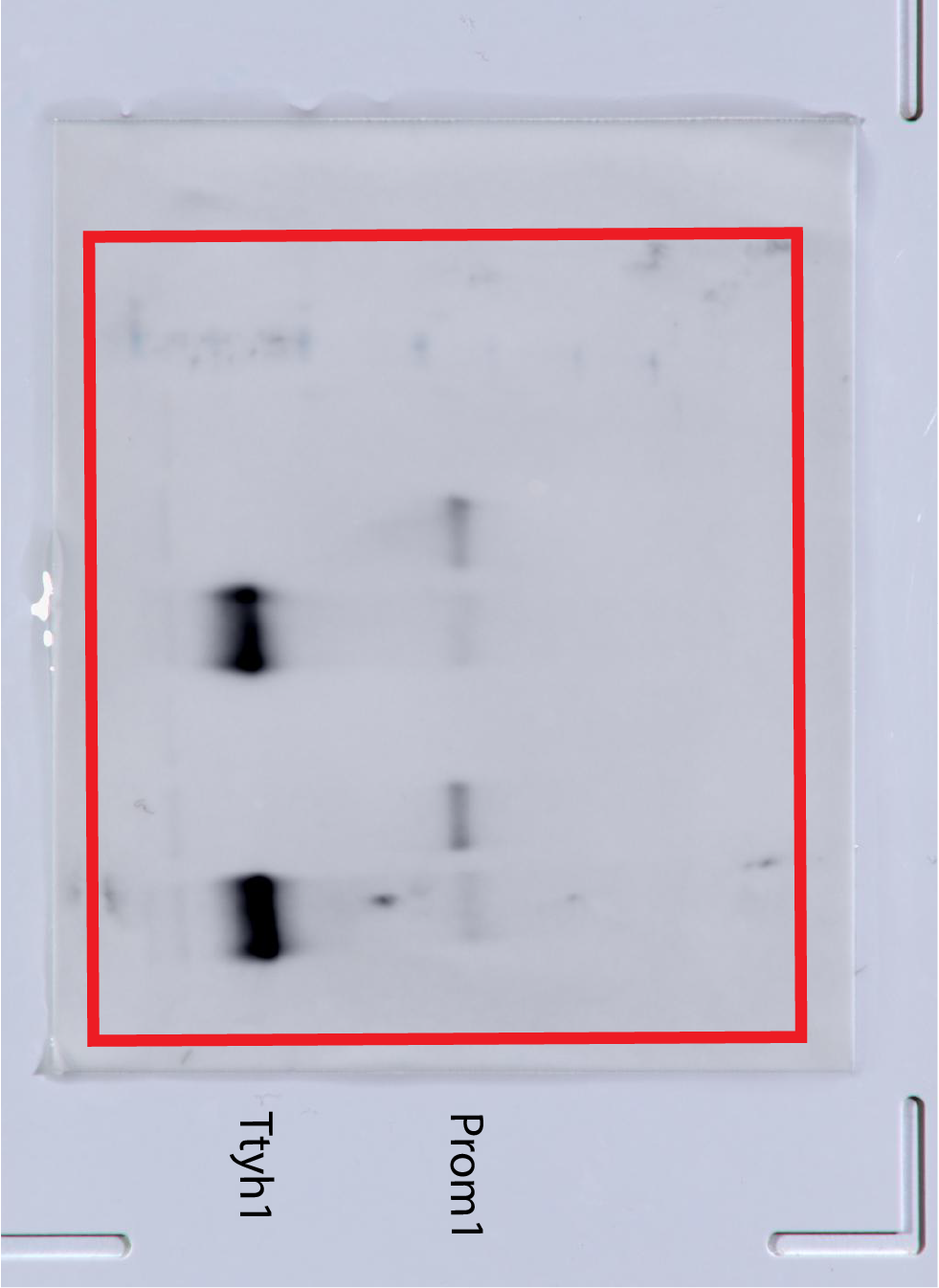

Supplement: Figure 3—figure supplement 1—source data 2. [file elife-100061-fig3-figsupp1-data2.zip › Figure 3-figure supplement 1-source data 2/Fig1B_left_WITH_RED_BOX_labeled.png]

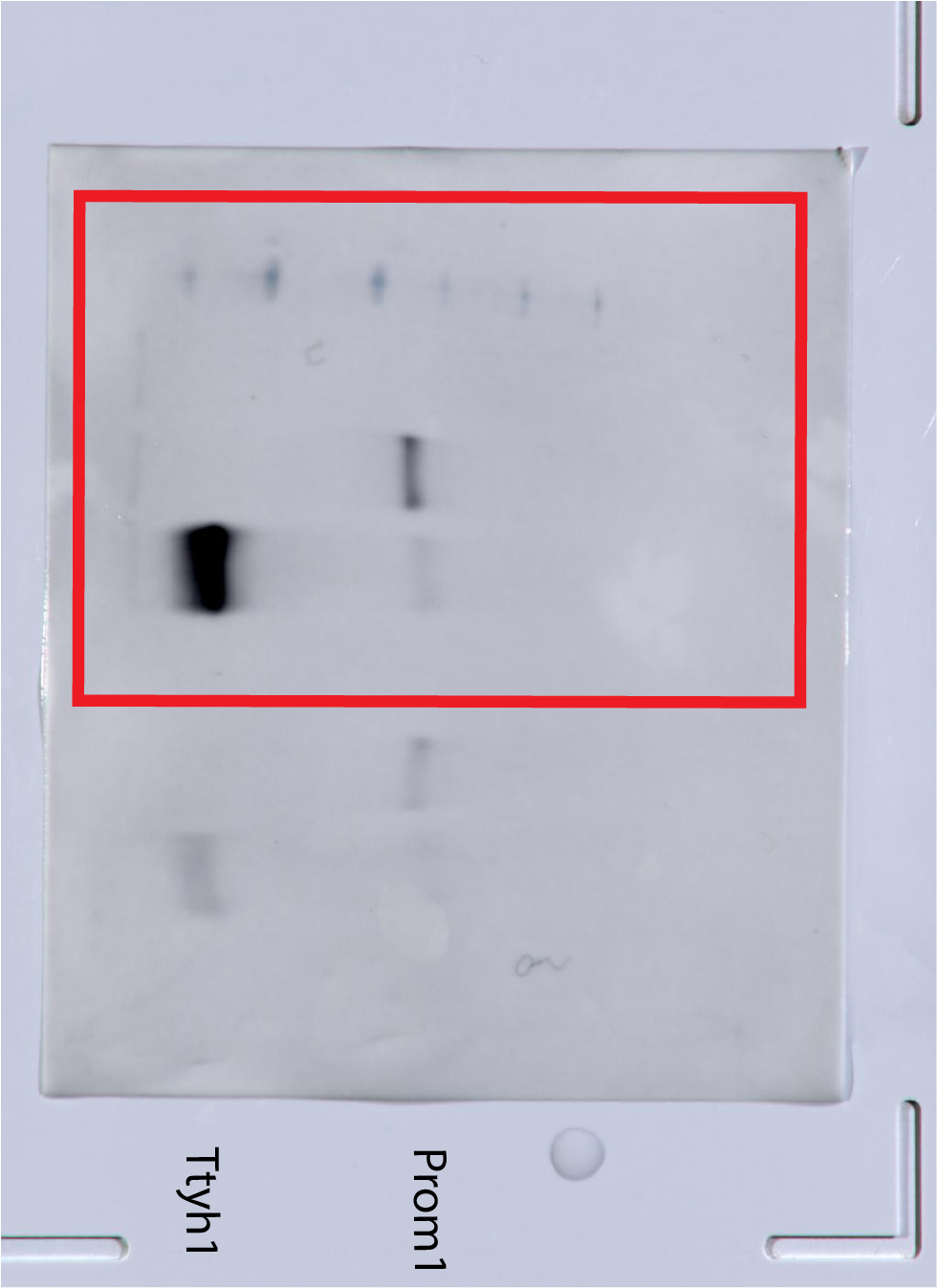

Supplement: Figure 3—figure supplement 1—source data 2. [file elife-100061-fig3-figsupp1-data2.zip › Figure 3-figure supplement 1-source data 2/Fig1B_right_WITH_RED_BOX_labeled.png]
