# Supplementary material for: Prominin 1 and Tweety Homology 1 both induce extracellular vesicle formation
Source: eLife. 2024 Aug 13;13:e100061. doi: 10.7554/eLife.100061 (PMC11405016; doi:10.7554/eLife.100061)
Supplement: Supplementary file 1. [file elife-100061-supp1.docx]

| **Prom1 Mutant** | **Specific Mutations** |
| --- | --- |
| Wild-type (WT) | - - |
| CRAC-1 | V460A, Y463L |
| CRAC-2 | Y819L |
| CRAC-3 | L125A, F130L |
| CRAC-4 | L806A, F811L |
| CARC-1 | F158L, L162A, L163A |
| CARC-2 | F804L, L806A, L809A |
| F62L | F62L |
| F505L | F505L |
| F794L | F794L |
| W795R | W795R |
| F796L | F796L |

**Supplementary table 1.** Prom1 mutants used in this study. All mutants are adapted from isoform S1 (NCBI accession NP_001139319.1).
